# Positive Effect of Acetylation on Proteomic Analysis Based on Liquid Chromatography with Atmospheric Pressure Chemical Ionization and Photoionization Mass Spectrometry

**DOI:** 10.3390/molecules28093711

**Published:** 2023-04-25

**Authors:** Simona Sedláčková, Martin Hubálek, Vladimír Vrkoslav, Miroslava Blechová, Petr Kozlík, Josef Cvačka

**Affiliations:** 1 Institute of Organic Chemistry and Biochemistry of the Czech Academy of Sciences, Flemingovo Náměstí 542/2, 16000 Prague, Czech Republic; simona.sedlackova@uochb.cas.cz (S.S.); martin.hubalek@uochb.cas.cz (M.H.); vladimir.vrkoslav@uochb.cas.cz (V.V.); miroslava.blechova@uochb.cas.cz (M.B.); 2 Department of Analytical Chemistry, Faculty of Science, Charles University, Hlavova 2030/8, 12800 Prague, Czech Republic; petr.kozlik@natur.cuni.cz

**Keywords:** acetylation, chemical ionization, photoionization, proteomics

## Abstract

A typical bottom-up proteomic workflow comprises sample digestion with trypsin, separation of the hydrolysate using reversed-phase HPLC, and detection of peptides via electrospray ionization (ESI) tandem mass spectrometry. Despite the advantages and wide usage of protein identification and quantification, the procedure has limitations. Some domains or parts of the proteins may remain inadequately described due to inefficient detection of certain peptides. This study presents an alternative approach based on sample acetylation and mass spectrometry with atmospheric pressure chemical ionization (APCI) and atmospheric pressure photoionization (APPI). These ionizations allowed for improved detection of acetylated peptides obtained via chymotrypsin or glutamyl peptidase I (Glu-C) digestion. APCI and APPI spectra of acetylated peptides often provided sequence information already at the full scan level, while fragmentation spectra of protonated molecules and sodium adducts were easy to interpret. As demonstrated for bovine serum albumin, acetylation improved proteomic analysis. Compared to ESI, gas-phase ionizations APCI and APPI made it possible to detect more peptides and provide better sequence coverages in most cases. Importantly, APCI and APPI detected many peptides which passed unnoticed in the ESI source. Therefore, analytical methods based on chymotrypsin or Glu-C digestion, acetylation, and APPI or APCI provide data complementary to classical bottom-up proteomics.

## 1. Introduction

Bottom-up proteomics is a widely used mass spectrometry method for identifying and quantifying proteins in complex mixtures, such as cell lysates [1,2]. A typical workflow starts with isolating proteins from a biological sample. The proteins are then enzymatically cleaved and the resulting peptide products are analyzed via HPLC/electrospray (ESI)-MS/MS. Search engines are finally used to match mass spectra with data from primary sequence databases. Although bottom-up proteomics is highly efficient and well-established, it has limitations. Some peptides may remain undetected, which reduces sequence coverage and the number of identified and quantified proteins. The factors limiting data quality can be traced in all steps of the analytical workflow. In the sample preparation, insufficient solubilization of highly hydrophobic proteins can hinder complete digestion and contribute to protein precipitation or adhesion to plastic surfaces [3,4,5]. Trypsin is a gold standard in proteomics; however, tryptic digestion of membrane proteins tends to reveal only peptides from soluble loops and terminal tails [6]. The enormous variability of protein properties and concentrations creates troubles during chromatographic separation. Highly hydrophobic peptides are strongly retained on reversed-phase columns; thus, they may not elute. In contrast, short hydrophilic peptides do not interact with the stationary phase sufficiently and are often lost in the void volume [7]. The separation efficiency can be further reduced for peptides with basic residues [8]. Electrospray is prone to matrix effects resulting from impurities from the sample, sample preparation, or chromatography (e.g., surfactants, plasticizers, salts, buffers, or ion-pairing reagents), which change the peptide signal intensities [9]. Residues of detergents used during sample preparation cause significant suppression of peptide signals [10]. MS/MS provides hundreds of spectra even for simple protein digests. Although search engine algorithms constantly improve, they may provide false positive or negative results due to the limited quality of spectra or inappropriate proteolytic cleavage.

Considerable progress is being made to overcome the limitations, especially for membrane proteins that are difficult to analyze due to their limited solubility in aqueous media. For such proteins, solubilization in methanol [11] or alternative detergents, including amphipols, fluorinated surfactants, fluorinated glucose, maltose-based detergents, and disulfide-containing amphiphiles [12,13,14,15], has been suggested. Protein identification and post-translational modifications (PTMs) detection can be improved via utilization of alternative proteases, such as chymotrypsin [6], glutamyl peptidase I (Glu-C) [16], pepsin [17], proteinase K [18], elastase [6] or chemical cleavage with cyanogen bromide [11]. New approaches in chromatographic separations of peptides are based on two-dimensional (2D) chromatography [19]. Retention of polar substances can be increased through matching the mobile phase pH to the peptide isoelectric point (pI). The approach is efficient; nevertheless, it is troublesome when detecting many peptides with variable pI values. Alternatively, ion-pairing reagents, such as alkylated fluorinated carboxylic acids (e.g., trifluoroacetic acid, trifluoro heptanoic acid, heptaflurorobutyric acid, or tridecafluoroheptanoic acid) [20] or alkylated sodium sulfates (e.g., hexyl sodium sulfate, octyl sodium sulfate) [21], can be used. Such reagents improve retention and peak shapes through forming neutral ion pairs and shielding cationic species from interaction with negatively charged silanols. However, significant suppression of the peptide signal occurs in the positive mode of electrospray ionization [22].

One of the possibilities for overcoming some of the limitations of bottom-up proteomics is the use of an alternative ionization technique. In addition to electrospray ionization, atmospheric pressure chemical ionization (APCI) [23,24] and photoionization (APPI) [25] have proved successful in many LC-MS applications. Ionization occurs via completely different mechanisms than in the case of electrospray ionization. In APCI, the cascade of gas-phase ionization reactions is initiated using electrons in the corona discharge plasma, while in APPI vacuum ultraviolet photons are used. The final ionization step is proton transfer, leading to mostly singly charged protonated molecules. The alkali metal or ammonium adduct ions forming more easily in APPI than APCI are attributed to thermospray ionization [26,27,28]. Multiply charged ions are rare in gas-phase ionizations [29]. APCI and APPI are less prone to matrix effects than electrospray ionization [30,31,32]. Both APCI and APPI have demonstrated the ability to detect peptides. APCI was previously efficiently utilized for peptides up to 600 Da [26], cyclic peptides [33], and iminodipeptides [34]. APPI proved effective for detecting hydrophobic peptides with a higher molecular weight [35,36], as well as PTMs such as palmitoylation [37,38]. In our recent study, both gas-phase ionization techniques efficiently detected peptides from Glu-C and chymotrypsin digests [39].

Chemical derivatization is widely used in proteomics to improve chromatographic behavior and detectability of peptides. Various structural motifs are targeted, including amino, carboxy, hydroxy, carbonyl, and thiol groups [40,41,42]. Derivatization of amino groups is carried out frequently to enhance de novo sequencing [43,44] or quantification [45,46] or reveal PTMs [45]. Many reagents, including *N*-hydroxysuccinimide (NHS)-esters, sulfo-NHS esters, aryl halides, fluorophenyl esters, and chloroformates, carry a permanent charge, which is beneficial for electrospray ionization. Introducing a permanent charge is not useful for gas phase ionizations, e.g., APCI [47]. Moreover, permanently charged molecules are more difficult to separate via reversed-phase chromatography [48].

Acetylation of amino groups is a fast, efficient, and well-established derivatization method that increases the hydrophobicity of analytes. The primary targets for the acetylation of proteins and peptides are ɛ amino groups in lysine residues and amino groups at the *N*-termini of the peptide chains. Other acids bearing amino groups in their side chains, such as arginine, glutamine, and asparagine, remain mostly unmodified. Acetylation was previously used to improve ionization efficiency in older methods, such as fast atom bombardment (FAB) mass spectrometry [49,50,51]. The higher [M + H]^+^ signals were linked to increased proton affinity (PA) and reduced hydrogen bonding to the FAB matrix [50,51]. The FAB spectra of acetylated peptides provided low-mass amino acid fragments, allowing the estimation of peptides’ amino acid composition [52]. Acetylation also improved the detection sensitivity of sterols in thermospray ionization [53]. The positive effect of acetylation was also observed with electrospray ionization [54,55,56,57]. Acetylated peptides showed improved fragmentation patterns, allowing for better sequence information [56]. The intensities of *b* [54,55] and *y* ions increased [56], while less informative fragments were suppressed. Acetylated peptides were used for de novo sequencing [55,57]. Acetylation is also used to improve analyte detection in APPI-MS [58]. Acetylated substance P and substance P in dopant APPI-MS provided full scan spectra with *c*, *b*, *y* ions and less abundant *a* ions [29]. Investigation of c-type fragmentations led to the proposal of electron transfer/electron capture dissociation mechanisms in APPI of peptides. LCAPCI-MS was used for analyzing acetylated amino acids in urine [59].

This work presents a study aimed at developing an alternative bottom-up proteomic workflow based on detecting acetylated peptides via HPLC coupled with APCI and APPI mass spectrometry. Mass spectra of acetylated peptides and intensities of peptide signals in various solvents were investigated. Two ways of achieving sample acetylation were compared: (i) derivatization at protein level; and (ii) derivatization at peptides level. Bovine serum albumin (BSA) and Glu-C or chymotrypsin digestion were used to show that sample acetylation, followed by APCI or APPI MS, is an efficient way of analyzing a proteomic sample.

## 2. Results and Discussion

### 2.1. Mass Spectra of Acetylated Peptide Standards

**Figure 1:**
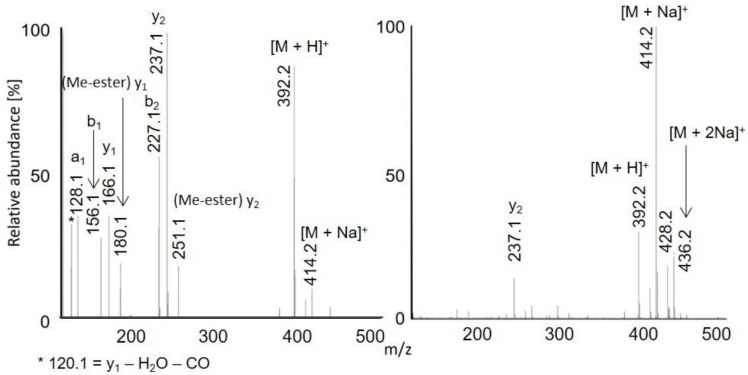
APCI (**left**) and APPI (**right**) full scan mass spectra of acetylated LAF. The peak marked with an asterisk is *m/z* 120.1, *y*1 − H_2_O − CO.

**Figure 2:**
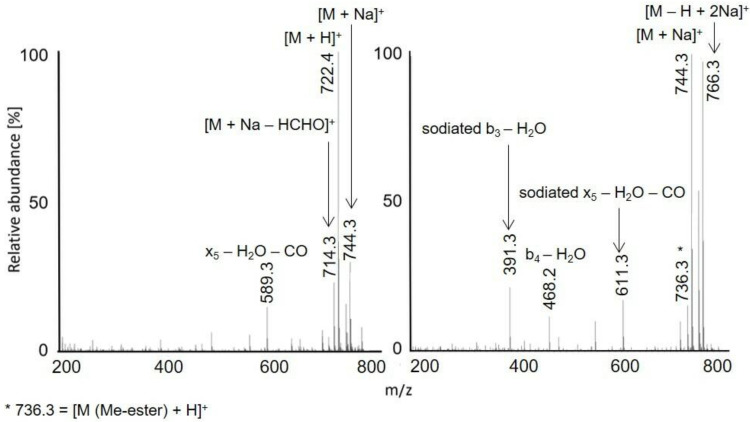
APCI (**left**) and APPI (**right**) full scan mass spectra of acetylated AWSVAF. The peak marked with an asterisk is *m/z* 736.3, [M (Me-ester) + H]^+^.

**Figure 3:**
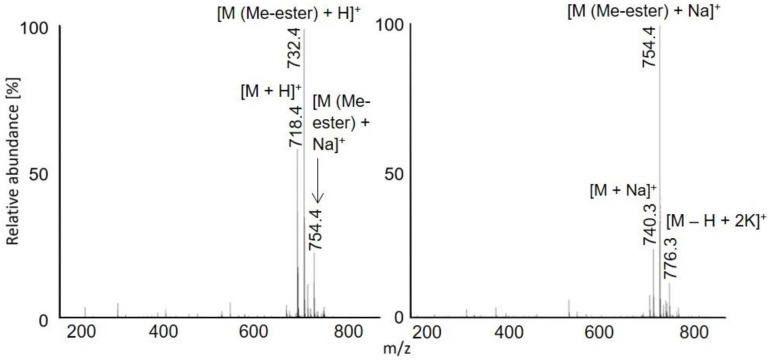
APCI (**left**) and APPI (**right**) full scan mass spectra of acetylated VLASSAE.

Full scan APCI and APPI mass spectra of acetylated peptides LAF, VASLF, SLGF, SLGE, SLGK, AWSVAF, VLASSAF, AWSVAE, VLASSAE, QTALVELLE, and QTALVELLF showed singly charged ions (Figure 1, Figure 2 and Figure 3, Figures S1, S4, S6, S9, S11, S13, S15 and S17). While APCI provided only protonated molecules, APPI spectra displayed protonated molecules and sodium adducts. All peptides were fully acetylated; no signals of non-acetylated species were detected. Free carboxy groups in acetylated peptides were partially esterified with methanol, which was used during the derivatization. Methyl esters were abundantly present in most samples of the peptides terminated with glutamic acid (VLASSAE, SLGE, and AWSVAE; Figure 3, Figures S1 and S11); significantly lower signals of methyl esters were detected in other samples. In-source fragmentation products were formed in both ionization modes and the fragments were more abundant for shorter peptides (Figure 1). The fragments were mostly *b* and *y* ions, as well as their H_2_O and NH_3_ loss products.

**Figure 4:**
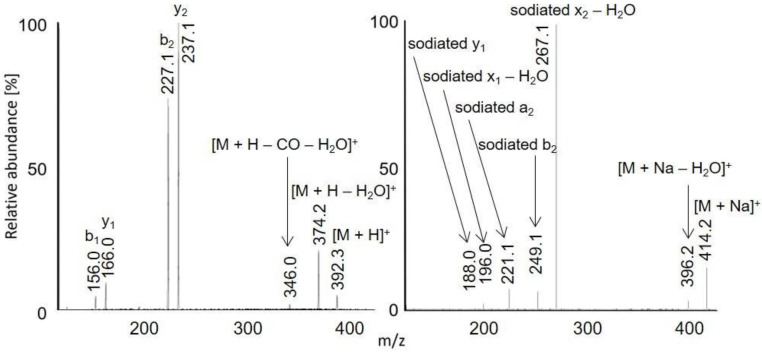
MS^2^ spectra of acetylated LAF: [M + H]^+^ *m/z* 392.2 in APCI (**left**) and [M + Na]^+^ *m/z* 414.2 in APPI (**right**).

**Figure 5:**
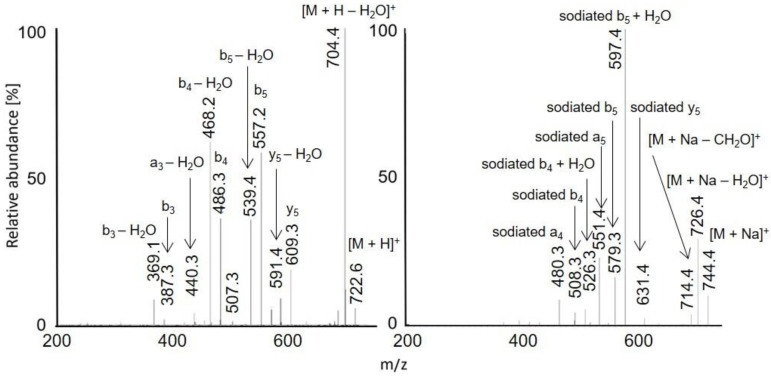
MS^2^ spectra of acetylated AWSVAF: [M + H]^+^ *m/z* 722.6 in APCI (**left**) and [M + Na]^+^ *m/z* 744.4 in APPI (**right**).

**Figure 6:**
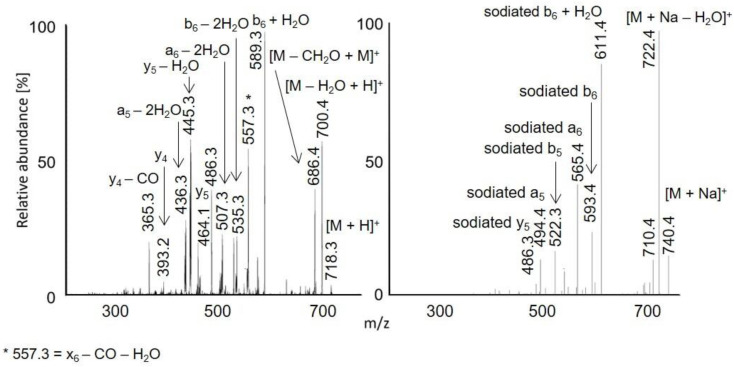
MS^2^ spectra of acetylated VLASSAE: [M + H]^+^ *m/z* 718.3 in APCI (**left**) and [M + Na]^+^ *m/z* 740.4 in APPI (**right**). The peak marked with an asterisk is *m/z* 557.3, *x*6 – CO − H_2_O.

Protonated molecules and sodium adducts of acetylated peptides and selected methyl esters of acetylated peptides were fragmented with CID in an ion trap. The fragmentation spectra of acetylated LAF, AWSVAF, and VLASSAE are shown in Figure 4, Figure 5 and Figure 6; mass spectra of other acetylated peptides are reported in the Supplementary Materials (Figures S2, S3, S5, S7, S8, S10, S12, S14, S16, and S18). Full scan and CID spectra were somewhat similar. Some fragments in the full scan spectra could be related to the thermal decomposition of the peptides.

The MS^2^ spectra of acetylated peptides differed depending on the type of precursor ions. Protonated and sodiated precursors provided abundant *b*, *y*, and *a* ions. In agreement with previous reports [60], MS^2^ spectra of acetylated peptides showed higher numbers and significantly more abundant *b* ions compared to non-acetylated peptides. Protonated peptides in low-energy CID produce *b* and *y* ion series. The *b* ions are less stable than *y* ions and may undergo cyclization in the gas phase; the subsequent ring opening leads to the formation of non-sequence fragments [61,62,63]. The non-sequence fragment ions complicate spectra interpretation and may cause erroneous conclusions. The extent to which the non-sequence peptide ions are detected depends on the peptides’ amino acid composition [63]. As the reaction is initiated via an amino group’s nucleophile attack of carbonyl, acetylation effectively prevents cyclization. Consequently, acetylated peptides provide simpler spectra with pronounced *b*-ion series and fewer non-sequence fragments [64]. Under low-energy conditions, unmodified peptides produce *b*1 ions only rarely [65,66,67], which can be improved via modifying their *N*-termini [68]. A recent study [60] confirmed that acetylation improves *b*1 detection for various peptides. The acetylated LAF provided abundant *b*1 ion *m/z* 156.1 in both full scan and MS^2^ APCI spectra (Figure 1 and Figure 2). Detecting *b*1 fragments in spectra of other peptides was impossible because of the ion trap’s low mass range cutoff. Besides common water loss products, *b* + *H*_2_*O* ions were observed frequently for sodiated peptide precursors (AWSVAF, VLASSAE, VASLF, QTALVELLF, AWSVAE, SLGF, SLGK, and VLASSAF). The *b* + *H*_2_*O* fragments can be formed from peptide ions with a charge at the N-terminus, allowing a rearrangement reaction that leads to the loss of the C-terminal residue [69,70]. This fragmentation pattern is often observed for alkali metal-cationized peptides [71]. The sodiated peptide precursors also yielded *a* ions, likely due to the loss of CO from *b* ions [72]. Regarding eliminating small neutral molecules from the peptide precursors, NH_3_ and H_2_O are typical losses for non-acetylated peptides [39]. Neutral loss of NH_3_ was rarely detected for acetylated peptides; instead, elimination of H_2_O, CO, and HCHO occurred. A neutral loss of CH_2_CO was observed for diacetylated SLGK (Figure S5).

The methyl esters of acetylated SLGE and VASLF showed a similar behavior as nonmethylated acetylated peptides, providing abundant *a*, *b*, and *y* ions in MS^2^ (Figures S3 and S8). Abundant molecular and fragment ions corresponding to neutral losses of water and HCHO were observed.

The effect of acetylation on peptide response was tested for VLASSAF and QTALVELLE. Flow injection analysis peaks reconstructed for [M + H]^+^ (APCI, ESI) or [M + Na]^+^ (APPI) were used to compare signals of acetylated and non-acetylated peptides. In APCI and APPI, acetylation improved the detection of the peptides. The ratio of peak heights for the acetylated and non-acetylated peptides in APCI was 3.0:1.0 (for both VLASSAF and QTALVELLE). The effect of acetylation was less pronounced in APPI; the acetylated and non-acetylated peptides ratio was 1.2:1.0 for VLASSAF and 2.2:1.0 for QTALVELLE. In ESI, acetylation did not affect the signal of VLASSAF; however, in the case of QTALVELLE, acetylation increased the intensity of singly charged molecular adduct almost two-fold (1.9:1.0).

### 2.2. Effect of the Mobile Phase on the Ionization of Acetylated Peptides

**Figure 7:**
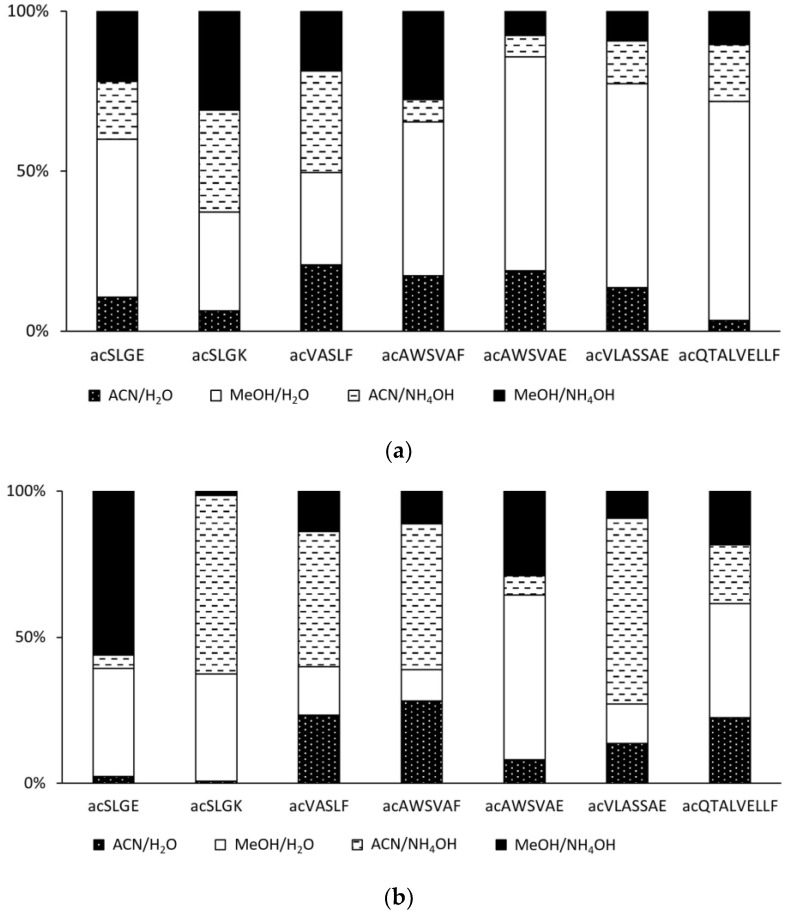
Normalized intensities of [M + H]^+^ peaks of acetylated peptide standards in various mobile phases for (**a**) APCI and (**b**) APPI.

The mobile phases for separating peptides usually contain acetonitrile or methanol and an aqueous buffer solution. In addition to providing separation, the mobile phase must also effectively support the ionization of the analytes. As regards gas-phase ionization methods, i.e., APCI and APPI, we have previously shown that ionization of peptides is most efficient when no buffer is present and the mobile phase is neutral or basic [39]. Here we tested the signals of acetylated SLGE, SLGK, VASLF, AWSVAF, AWSVAE, VLASSAE, and QTALVELLF in acetonitrile and methanol combined with water or aqueous ammonium hydroxide (pH 9). The organic and aqueous phase ratio was 4:1 (*v/v*). The relative intensities of peptide signals varied; however, all mobile phase combinations proved efficient for gas-phase ionization (Figure 7). In APCI, five out of seven tested peptides provided significantly higher signals in the aqueous mobile phases with no pH adjustment. In APPI, however, most peptides were efficiently ionized in the ammonia-treated mobile phase. The difference between methanol and acetonitrile can be related to the proton affinities of these solvents and the analyzed peptides. Methanol has lower PA than acetonitrile [73], facilitating efficient ionization of peptides with a relatively low PA.

### 2.3. Bottom-Up Proteomics of BSA Digests

BSA digests were used to test the applicability of acetylation in bottom-up proteomic workflows based on APCI and APPI. The digests were prepared using chymotrypsin or Glu-C, which were shown to provide peptides with better properties than trypsin for gas-phase ionizations [39].

Two different ways of acetylation were compared. In the first case, the protein was acetylated before its enzymatic digestion. Acetylation occurred on ε amino groups of lysines and at the protein N-terminus. In the second approach, acetylation of digested peptides was performed, yielding peptides with modified lysines and N-termini. The third set of samples were enzymatic digests of unmodified BSA. The peptide samples were analyzed via HPLC/MS^2^ with ESI, APCI, and APPI sources.

The absence of unmodified peptides in the acetylated protein digests indicated a high derivatization efficiency. Acetylation occurred exclusively on lysine side chains and N-termini; no acetylation on other amino acids was detected. Due to the presence of methanol during the derivatization, a small amount of ester by-products was formed. As with the peptide standards, APCI and APPI provided singly charged protonated molecules and fragments, while sodiated molecules were readily formed in APPI. ESI showed singly and multiply charged peptide ions. The HPLC/MS^2^ data were processed using the Mascot search engine to identify peptides and evaluate sequence coverage.

After acetylation, sequence coverage increased, regardless of the method used for ionizing peptides (Table 1). In APCI, a moderate improvement in sequence coverage was observed for samples acetylated at protein and peptide levels. While unmodified peptides made it possible to describe 21–22% of the BSA sequence, acetylation improved the value by up to half. ESI provided high sequence coverage values, though the effect of acetylation was less important than in the case of APCI-MS. The highest values of sequence coverage were achieved in APPI for acetylated samples. Acetylation at the protein level increased the value from 22 to 34% for chymotrypsin and from 42 to 54% for Glu-C digestion. Acetylation of peptides resulted in an even more significant increase in sequence coverage to 43% and 62% for chymotrypsin and Glu-C digestion, respectively. The complete list of detected peptides is included in the Supplementary Materials (Tables S1–S6).

In APCI, acetylation improved the number of peptides detected in the chymotrypsin digest (Table 2). Although the number of acetylated peptides was relatively high, most of them were rather short; about 70–80% consisted of six or fewer amino acids. Half of the peptides detected via APCI in Glu-C digests were also short. Therefore, APCI demonstrated high sensitivity towards short peptides [26]. Data were also consistent with the nature of enzyme cleavages, i.e., the ability of chymotrypsin to form shorter peptides compared to Glu-C. In the case of BSA, chymotrypsin theoretically provides 55 short peptides (6 or fewer amino acids) from a total of 95 peptides, while Glu-C produces 39 short peptides from 86 peptides. Few methyl esters of acetylated peptides were detected; these were mostly detected in the sample derivatized at the peptides level, where the C-termini of the peptides were exposed to the reagent. In this sample, seven of 21 detected acetylated peptides were methylated at a significant level. Peptides with methylated carboxyl groups were also observed at comparable levels in APPI and ESI.

In APPI, acetylation did not increase the number of detected peptides in the chymotrypsin digest but made detecting more peptides with longer sequences possible (Table 3). As for the Glu-C digest, acetylation helped to increase the number of detected peptides significantly. This was attributed to a reduction in the polarity of the peptides which, thus, transferred more easily to the gas phase. APPI detected a higher number of longer peptides than APCI, particularly in the Glu-C digest. Sodium adducts were formed in APPI; approximately half of the acetylated peptides were detected as sodium adducts. The singly charged sodium adducts provided sequence-specific information [71] and improved the sensitivity compared to protonated molecules [74,75].

In ESI, similar sequence coverages and numbers of detected peptides were achieved for acetylated and non-acetylated digests. However, the structures of the individual peptides differed. The detection of different peptides can be explained using two factors influencing ionization. On one hand, increasing the hydrophobicity leads to easier migration of the peptides to the surface of the droplets, thus improving ionization efficiency [76,77,78]. On the other hand, decreasing the basicity of lysines leads to a lower ability to accept a proton. These two contending events had a different combined effect on various peptides (Table 4). The protein digests provided singly, doubly, triply, and quadruply charged ions. In the non-acetylated sample, doubly charged ions were mainly detected and singly and triply charged ions were formed less efficiently. As regards the acetylated samples, singly charged ions were formed the most, followed by doubly and, exceptionally, triply charged ions. Neutralization of the charge on lysines reduced the charge states of the peptides. For example, the non-derivatized form of DKLKHLVDEPQNL was detected as a 3+ ion, singly acetylated as 2+, and doubly acetylated as 1+. A charge state reduction resulting from acetylation was also observed for DEHVKL, GERAL, KDLGEEHF, QEAKDAF, SQKFPKAEF, FKADE, RMPCTE, CCHGDLLE, and LCKVASLRE. As shown in Table 4, multiply-charged peptides comprised about 70% of the peptides in the BSA digest but fewer than 50% in acetylated samples.

**Figure 8:**
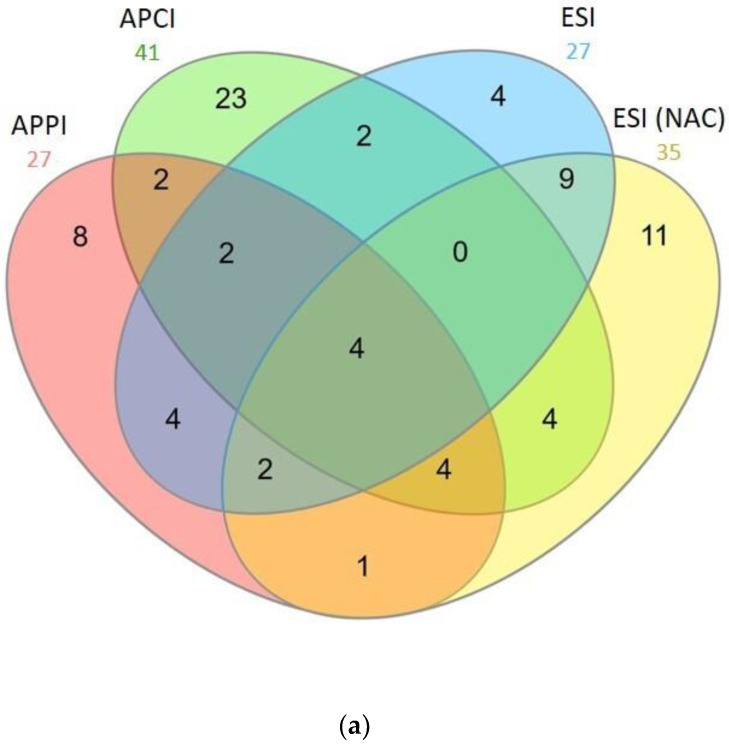

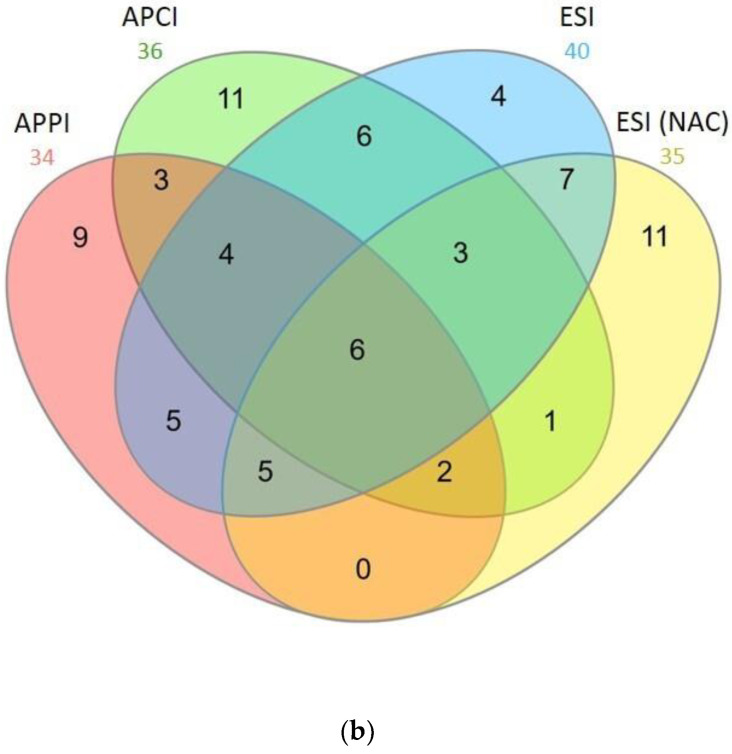
Venn’s diagram with number of peptides detected in BSA chymotrypsin digests using APPI, APCI, and ESI-MS. Sample was acetylated at protein level (upper diagram, (**a**) and peptide level (lower diagram, (**b**). ESI data for non-acetylated sample (NAC) are shown for comparison. BSA peptides (10 μL injected; 0.65 mmol·L^−1^) were separated at 50 °C using a 20 min gradient and mobile phase flow rate was 200 μL·min^−1^. Data were evaluated using Mascot engine.

**Figure 9:**
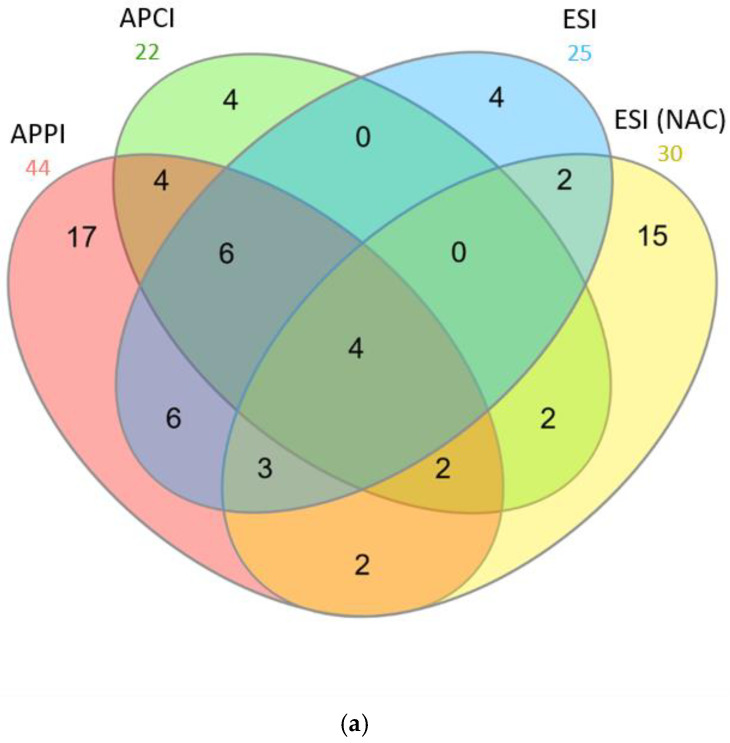

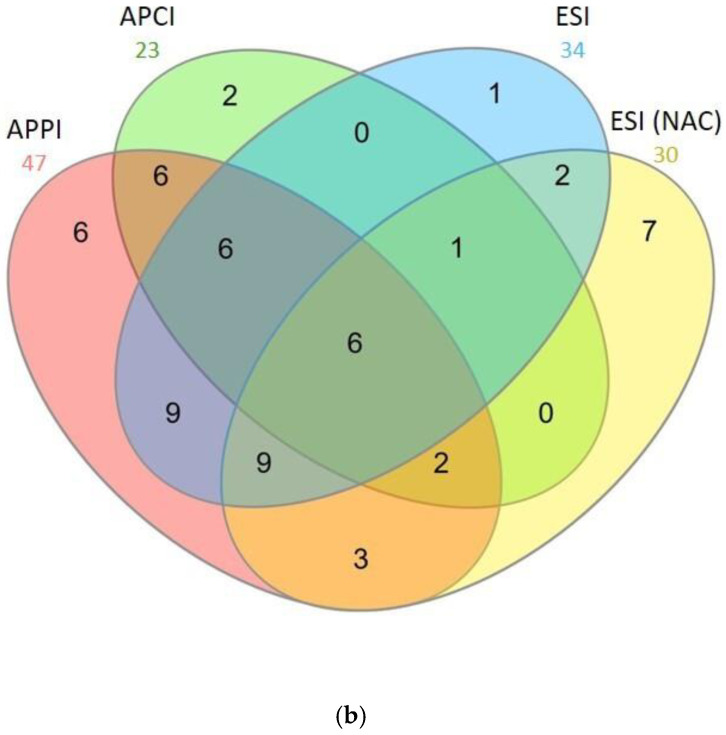
Venn’s diagram with number of peptides detected in BSA Glu-C digests using APPI, APCI, and ESI-MS. Sample was acetylated at protein level (upper diagram, (**a**) and peptide level (lower diagram, (**b**). ESI data for non-acetylated sample (NAC) are shown for comparison. The BSA peptides (10 μL injected; 0.65 mmol·L^−1^) were separated at 50 °C by a 20 min gradient and mobile phase flow rate was 200 μL·min^−1^. Data were evaluated using Mascot engine.

APPI, APCI, and ESI detected unique peptides in acetylated chymotrypsin and Glu-C digests. The most powerful ionization regarding the number of total and unique peptides in the chymotrypsin digest was APCI (Figure 8). However, the peptides were mostly short, meaning that the sequence coverage was unsatisfactory. On the contrary, the ionization that performed best for the Glu-C digest was APPI (Figure 9). APPI made it possible to detect more peptides, including many unique ones, than other ionizations. The detected peptides were mostly of high molecular weight (more than six amino acids). Workflows involving acetylation, Glu-C cleavage, and detection through APPI-MS also gave the best results in terms of sequence coverage (Table 1). The superior performance of APPI-MS for Glu-C digest can be related to the easier formation of sodium adducts of E- and D-terminated peptides. APPI-MS also easily detected longer acetylated peptides formed through Glu-C.

The detection of unique peptides can be utilized in novel proteomic workflows based on combining different ionization techniques. Such workflows describe primary protein sequences more comprehensively than the classical bottom-up approach. To test the hypothesis, we combined ESI, APCI, and APPI data to calculate sequence coverages (Table 5). The classical bottom-up proteomics based on ESI allowed us to describe 37% of the BSA sequence (Table 1). APCI made it possible to reveal an additional ~10% of the sequence. When ESI data were combined with APPI, sequence coverage increased by ~20% (chymotrypsin digest) and ~30% (Glu-C digest). Thus, APCI and APPI have yielded considerable complementary information to the classical ESI-based workflow. In addition to the ionization techniques, combining two or more proteases could further increase the number of detected peptides and sequence coverages [79].

The use of APCI and APPI in proteomics is limited due to the minimum flow rates that can be used with these ion sources. Commercial sources are designed for high mobile phase flow rates and operate optimally at around 1 mL·min^−1^. Nevertheless, some of the ion sources make it possible to reduce the flow rate down to 50 μL·min^−1^ and, thus, enable APCI and APPI MS detection in microflow HPLC. The use of commercial APCI and APPI sources with capillary or nanoflow HPLC is not possible; however, ion sources dedicated to capillary or nanoflow HPLC are being developed [80,81,82,83].

## 3. Materials and Methods

### 3.1. Chemicals and Reagent

Ammonium formate (reagent grade; 97%), ammonium bicarbonate (reagent grade), and bovine serum albumin (BSA; ≥98%) were purchased from Fluka Biochemica (Buchs, Switzerland). Trifluoroacetic acid (TFA) for HPLC (>99.9%), acetic anhydride (≥97%), ammonia solution (25%) for LC-MS, iodoacetamide (IAA; reagent grade), dithiothreitol (DTT; reagent grade), and triisopropylsilane (TIS; 98%) were obtained from Sigma Aldrich (St. Louise, WA, USA). Formic acid (FA; 98%) and acetic acid (99%) were purchased from Lach-Ner (Neratovice, Czech Republic). Methanol and acetonitrile (both OPTIMA LC-MS) were obtained from Fisher Chemical (Waltham, MA, USA). Sequencing grade chymotrypsin and Glu-C were purchased from Roche Diagnostics (Mannheim, Germany). Fmoc Arg(Pbf) WANG resin, Fmoc Lys(Boc) WANG resin, Fmoc Phe WANG, and Fmoc Glu(OtBu) WANG for peptide standards synthesis were purchased from Iris Biotech (Marktredwitz, Germany).

### 3.2. Peptide Standards

Peptide standards SLGK, SLGE, AWSVAE, VLASSAE, QTALVELLE, LAF, SLGF, VASLF, AWSVAF, VLASSAF, and QTALVELLF (all with free carboxyl on C-terminus) were synthesized in house. The peptides corresponded to BSA tryptic products and analogs with phenylalanine or glutamic acid at C-terminus, i.e., hypothetical peptides that could be formed using chymotrypsin or Glu-C cleavage. Peptide sequences were assembled on a Liberty Blue solid-phase synthesizer from CEM (Charlotte, NC, USA) through stepwise coupling of the corresponding Fmoc-amino acids to the growing chain on a Fmoc-Arg(Pbf)-WANG resin (100–200 mesh, 0.64 mmol·g^−1^); Fmoc-Lys (Boc)-WANG resin (100–200 mesh, 0.65 mmol·g^−1^); FmocGlu(OtBu)-WANG resin (200–400 mesh, 0.62 mmol·g^−1^), and Fmoc-Phe-WANG resin (200–400 mesh, 0.67 mmol·g^−1^). Fully protected peptides were synthesized according to a standard procedure involving cleavage of the Nα-Fmoc protecting group with 20% piperidine in dimethylformamide (DMF) (HPLC grade) and coupling, mediated with mixtures of coupling reagents (diisopropyl carbodiimide) DIC/(oxyma-ethylcyanohydroxyiminoacetate) Oxyma in DMF. On completion of synthesis, the deprotection and detachment of the linear peptides from the resins were carried out simultaneously using a TFA/H_2_O/TIS (95.0:2.5:2.5) cleavage mixture. Each resin was washed with dichloromethane and the combined TFA filtrates were evaporated at room temperature. The precipitated residues were triturated with *tert*-butyl methyl ether, collected via suction, and dried via lyophilization. The linear peptides were purified via HPLC using a Waters instrument with Delta 600 pump and a 2489 UV/VIS detector.

### 3.3. Protein Digestion

Proteins were digested using the enhanced filter-aided sample preparation (eFASP) method [84]. Protein dissolved in 80% acetonitrile 0.1% FA (125 μL; 1 mg·mL^−1^) was diluted with ammonium bicarbonate (50 mmol·L^−1^; pH 7.8) to a volume of 200 μL. The reduction was initiated by adding 15 μL of DTT (100 mmol·L^−1^ in water) and the sample was incubated for 30 min at 65 °C. Free thiol groups were alkylated with 45 μL of IAA (100 mmol·L^−1^ in water) and the sample was incubated in the dark for 30 min at 20 °C. The alkylation was terminated by adding 30 μL of DTT (100 mmol·L^−1^ in water). The sample was washed three times with 100 μL of 50 mmol·L^−1^ ammonium bicarbonate on Microcon centrifugal filters (Ultracel PL 10, Merck, Ireland) using Eppendorf Centrifuge 5417 Refrigerator (Boston, MA, USA). The digestion was performed on the same filter in 100 μL of 50 mmol·L^−1^ ammonium bicarbonate in the presence of protease (0.1 mg·mL^−1^) in a weight ratio of (chymotrypsin or Glu-C: protein) 1:50. Digestion of protein was performed with chymotrypsin at 30 °C and Glu-C for 16 h at 20 °C. Protein digest was washed twice with 75 μL of 50 mmol·L^−1^ ammonium bicarbonate via centrifugation and the filtrate was evaporated to dryness on a refrigerated centrifugal vacuum concentrator Labconco (Kansas City, MI, USA).

### 3.4. Acetylation

A synthetic peptide standard was dissolved in 80% ACN 0.1% FA (1 mg·mL^−1^). To 20 μL of the standard solution, 50 μL of acetylation reagent (acetic anhydride and methanol *v/v* 1:3) was added. Acetylation was carried out in an Eppendorf tube over 1 h at 25 °C in a thermoshaker. Acetic anhydride with methanol was evaporated on a refrigerated centrifugal vacuum concentrator.

Two approaches were used to acetylate BSA. Acetylation at the protein level caused the acetylation of lysine ɛ amines and protein N-termini. BSA was dissolved in 80% acetonitrile 0.1% FA (125 μL; 1 mg·mL^−1^), protein disulfide bonds were reduced via DTT, and thiol groups were alkylated via IAA according to the procedure described in Section 3.3. After the complete alkylation, 50 μL of the acetylating agent was added to 20 μg of BSA and the acetylation was achieved as described above for peptide standards. After the complete acetylation, the sample was washed three times with 100 μL of 50 mmol·L^−1^ ammonium bicarbonate on Microcon centrifugal filters using Eppendorf Centrifuge and the digestion proceeded as described in Section 3.3.

Acetylation at the peptide level generated peptides acetylated on lysine side chains and N-termini amino groups. BSA was processed according to the digestion protocol described in Section 3.3. Subsequently, the sample was re-dissolved in 80% acetonitrile 0.1% FA (125 μL; 1 mg·mL^−1^) and acetylated in the same way as peptide standards.

### 3.5. Mass Spectrometry

The experiments were performed on an LTQ Orbitrap XL (Thermo Scientific, Waltham, MA, USA) that combines a linear ion trap MS and an Orbitrap mass analyzer. The mass spectrometer was equipped with an IonMax source operating in ESI, APCI, or APPI mode. Full scan mass spectra were collected using the Orbitrap operated at a resolution of 30,000. Collision-induced dissociation (CID) and fragments detection were performed in the ion trap. The optimum normalized collision energy (NCE) values varied depending on the peptide structure. For acetylated peptides, NCEs of 20–30% were used. The isolation width for fragmentations was set to 1 Da.

The mass spectrometer was operated in the positive ion mode. In ESI, the ion source parameters were as follows: sheath and auxiliary gases were 35 and 5 arbitrary units, respectively; the source voltage was 4.80 kV; the capillary voltage was 7.0 V; the tube lens offset was 100.0 V; and the capillary temperature was 250 °C. In APCI, sheath and auxiliary gases were set at 50 and 10 arbitrary units, respectively; probe temperature was 480 °C; the corona discharge current was 10.00 µA; the, tube lens offset was 110.0 V; and the capillary temperature was 275 °C. In APPI, a krypton lamp from Syagen Technology, Inc. (Tustin, CA, USA) that emits photons at 10.0 eV and 10.6 eV (123.6 nm and 118.0 nm) was used. The sheath and auxiliary gases were at 90 and 10 arbitrary units, respectively; the probe temperature was 480 °C; the tube lens offset was 160 V; and the capillary temperature was 275 °C.

### 3.6. Flow Injection Analysis

The relative responses of acetylated and non-acetylated VLASSAF (41 μmol·L^−1^) and QTALVELLE (28 μmol·L^−1^) were examined via flow injection analysis. The peptide standards (2.5 μL) were injected into the mobile phase consisting of methanol and water (4:1, *v/v*; flow rate of 200 μL·min^−1^). Peak heights were obtained from data reconstructed for [M + H]^+^ or [M + Na]^+^ with a 0.2 Da extraction window.

### 3.7. Liquid Chromatography-Tandem Mass Spectrometry

The mass spectrometer (Section 3.5) was coupled to a liquid chromatograph consisting of a Rheos 2200 LC-MS Pump (Thermo Scientific/Flux Instruments), HTS PAL Autosampler (CTC Analytics, Zwingen, Switzerland), and DeltaChrom CTC 100 column thermostat (Watrex, Prague, Czech Republic). The analyses of protein digest (650 μmol·L^−1^, 10 μL injected) were performed on an Acquity UPLC BEH C18 reversed-phase column (1.7 μm; 2.1 × 50.0 mm, Waters) maintained at 50 °C. The mobile phases were prepared from solvent A (water or water adjusted to pH 9 with ammonia), solvent B (methanol), and solvent C (acetonitrile) as follows: sample loading 0–1.0 min (4.0% of B; 1.0% of C) continued via linear gradients 1.0–5.0 min (4.0–24.0% of B; 1.0–6.0% C), 5.0–10.0 min (24.0–25.5% of B; 6.0–6.5% of C), 10.0–12.5 min (25.5–76.5% of B; 6.5–18.5% of C), 12.5–16.5 min (76.5%–4.0 of B, 18.5–1.0% of C), and 17.5–20.0 min (4.0 of B; 1.0% of C). The mobile phase flow rate was 200 μL·min^−1^.

The data-dependent scanning with four scan events was set as follows: the NCE was 35%, the dynamic exclusion was 60 s with the exclusion list size limited to 500 precursor ions, the signal threshold was 75 counts, the isolation width was 3 Da, and the activation time was 30.0 ms.

### 3.8. Data Interpretation

MS data were processed in Xcalibur (version 4.1, Thermo Fisher Scientific, Waltham, MA, USA) using the Mascot search engine (Version 2.2, [85]). The search parameters were set as follows: In MS^1^, the mass tolerance was defined at 10 ppm, while in MS^2^ it was defined at 0.6 Da. Carbamidomethylation of C, deamidation of N and Q, and oxidation of M for non-derivatized peptides were selected as dynamic modifications. In the case of modified peptides, acetylation was either only on lysine residues or also on the N-termini of the peptides, depending on the method of the experiment. Furthermore, methylations of C termini were detected in some cases and, especially with APPI, sodium adducts at C-termini and D or E were detected.

## 4. Conclusions

This work shows the advantages of acetylation for proteomic analysis based on APCI and APPI mass spectrometry. Acetylated peptides provided many sequence-specific *a*, *y*, and *b* fragments, allowing for straightforward data interpretation. The applicability of acetylation in bottom-up proteomics was investigated in workflows encompassing derivatization of intact BSA or BSA digests. The enzymatic cleavage was accomplished with chymotrypsin or Glu-C. The acetylation targeted amino groups of lysines and N-termini; other functional groups remained unmodified. The only exception was the occasional formation of methyl esters on C-termini via a reaction with methanol in the reaction mixture.

In the experiments with non-derivatized and acetylated samples, sequence coverage and numbers of peptides detected using APCI, APPI, and ESI were compared. The most pronounced effect of acetylation was observed in APPI, particularly for a sample cleaved with Glu-C and acetylated at the peptides level. In APCI, acetylation led to a moderate increase in sequence coverage and significant improvement in the detection of short peptides. Peptides detected via ESI, APCI, and APPI overlapped only partially; all ionization methods generated unique peptides. The workflows based on acetylation and gas-phase ionizations represent complementary approaches to ESI-based methods. APCI and APPI offer access to missing pieces of information on proteomes, which are difficult to analyze via the conventional bottom-up approach.

Due to its robustness, reproducibility, and good sensitivity, micro-flow LC–MS^2^ is increasingly used in many proteomic applications [86]. As some APCI and APPI sources are compatible with microflow systems, including these ionizations into HPLC/MS^2^, proteomic protocols are suggested. Using gas-phase ionizations will make it possible to detect peptides that are difficult to analyze using electrospray and advance the possibilities of MS-based proteomics. The possibility of using APCI and APPI even in capillary HPLC can be expected in the future; the development of ion sources dedicated to low mobile phase flow rates is the subject of research [80,81,82,83].

## Figures and Tables

**Table 1:** Sequence coverages for BSA chymotrypsin and Glu-C digests analyzed via HPLC/MS^2^ with APCI, APPI, or ESI sources. Sample was acetylated at protein or peptide level. BSA digests (10 μL injected; 0.65 mmol·L^−1^) were separated at 50 °C using a 20 min gradient elution. Mobile phase consisted of water (APCI) or water adjusted to pH 9 with ammonia (ESI, APPI), methanol, and acetonitrile; flow rate was 200 μL·min^−1^. Data were evaluated using the Mascot engine.

Ion Source	Protease	Sequence Coverage [%]		
		Acetylation at the Protein Level	Acetylation at the Peptides Level	No Acetylation
APCI	Chymotrypsin	30	25	21
	Glu-C	25	28	22
APPI	Chymotrypsin	34	43	22
	Glu-C	54	62	42
ESI	Chymotrypsin	40	40	37
	Glu-C	39	44	37

**Table 2:** Peptide types detected in BSA chymotrypsin and Glu-C digests via HPLC/MS^2^ with APCI source.

Protease	Type of Peptides	Number of Peptides		
		Acetylation at the Protein Level	Acetylation at the Peptides Level	No Acetylation
Chymotrypsin	All	41	36	29
	Acetylated methyl esters	1	1	0
	Long peptides *	7	8	7
	Sodium adducts	0	0	0
Glu-C	All	22	23	21
	Acetylated methyl esters	2	7	0
	Long peptides *	11	16	13
	Sodium adducts	0	0	0

* Peptides with more than six amino acids.

**Table 3:** Peptide types detected in BSA chymotrypsin and Glu-C digests via HPLC/MS^2^ with APPI source.

Protease	Types of Peptide	Number of Peptides		
		Acetylation at the Protein Level	Acetylation at the Peptides Level	No Acetylation
Chymotrypsin	All	27	34	30
	Acetylated methyl esters	2	0	0
	Long peptides *	17	19	6
	Sodium adducts	14	16	0
Glu-C	All	44	47	36
	Acetylated methyl esters	2	10	0
	Long peptides *	36	39	28
	Sodium adducts	21	26	4

* Peptides with more than six amino acids.

**Table 4:** Peptide types detected in BSA chymotrypsin and Glu-C digests via HPLC/MS^2^ with ESI source.

Protease	Type of Peptides		Number of Peptides	
		Acetylation at the Protein Level	Acetylation at the Peptides Level	No Acetylation
Chymotrypsin	All	27	40	35
	Acetylated methyl esters	3	4	0
	Long peptides *	20	23	23
	Sodium adducts	3	1	2
	Singly charged	15	28	11
	Doubly charged	10	10	15
	Triply charged	2	2	8
	Quadruply charged	0	0	1
Glu-C	All	25	34	30
	Acetylated methyl esters	2	6	0
	Long peptides *	21	24	23
	Sodium adducts	0	0	3
	Singly charged	15	20	7
	Doubly charged	10	14	18
	Triply charged	0	0	5
	Quadruply charged	0	0	0

* Peptides with more than six amino acids.

**Table 5:** Sequence coverages for BSA chymotrypsin and Glu-C digests analyzed via HPLC/MS^2^ and calculated via combining data from ESI, APCI, and APPI experiments. Sample for APCI and APPI was acetylated at protein or peptide level. BSA digests (10 μL injected; 0.65 mmol·L^−1^) were separated at 50 °C using a 20 min gradient elution. The mobile phase consisted of water (APCI) or water adjusted to pH 9 with ammonia (ESI, APPI), methanol, and acetonitrile; flow rate was 200 μL·min^−1^. Data were evaluated using Mascot engine.

Protease	Sequence Coverage [%]			
	Acetylated at the Protein Level *		Acetylated at the Peptides Level *	
	ESI (NAC) + APCI	ESI (NAC) + APPI	ESI (NAC) + APCI	ESI (NAC) + APPI
Chymotrypsin	49	56	47	54
Glu-C	48	70	49	71

* The sample for ESI was not acetylated.

## Data Availability

The data presented in this study are available on request from the corresponding author.

## Supplementary Materials

The following supporting information can be downloaded at: https://www.mdpi.com/article/10.3390/molecules28093711/s1, Figure S1. FTMS spectra of acetylated SLGE. Figure S2. MS^2^ spectra of acetylated SLGE. Figure S3. MS^2^ spectra of acetylated SLGE. Figure S4. FTMS spectra of acetylated SLGK. Figure S5. MS^2^ spectra of acetylated SLGK. Figure S6. FTMS spectra of acetylated VASLF. Figure S7. MS^2^ spectra of acetylated VASLF. Figure S8. MS^2^ spectra of acetylated VASLF. Figure S9. FTMS spectra of acetylated QTALVELLF. Figure S10. MS^2^ spectra of acetylated QTALVELLF. Figure S11. FTMS spectra of acetylated AWSVAE. Figure S12. MS^2^ spectra of acetylated AWSVAE. Figure S13. FTMS spectra of acetylated SLGF. Figure S14. MS^2^ spectra of acetylated SLGF. Figure S15. FTMS spectra of acetylated VLASSAF. Figure S16. MS2 spectra of acetylated VLASSAF. Figure S17. FTMS spectra of acetylated QTALVELLE. Figure S18. MS^2^ spectra of acetylated QTALVELLE. Table S1. Peptides detected via APPI in BSA digested using chymotrypsin. Table S2. Peptides detected via APCI in BSA digested using chymotrypsin. Table S3. Peptides detected via ESI in BSA digested using chymotrypsin. Table S4. Peptides detected via APPI in BSA digested using Glu-C. Table S5. Peptides detected via APCI in BSA digested using Glu-C. Table S6. Peptides detected via ESI in BSA digested using Glu-C.

## Sample Availability

Samples of the compounds are not available from the authors.
