# Supplementary material for: Positive Effect of Acetylation on Proteomic Analysis Based on Liquid Chromatography with Atmospheric Pressure Chemical Ionization and Photoionization Mass Spectrometry
Source: Molecules. 2023 Apr 25;28(9):3711. doi: 10.3390/molecules28093711 (PMC10180487; doi:10.3390/molecules28093711)
Supplement: Supplementary file 1 [file molecules-28-03711-s001.zip › molecules-2338505-supplementary.pdf]

Supplementary Materials

Positive Effect of Acetylation on Proteomic Analysis Based on Liquid Chromatography with Atmospheric Pressure Chemical Ionization and Photoionization Mass Spectrometry

Simona Sedláčková[1,2], Martin Hubálek[1], Vladimír Vrkoslav[1], Miroslava Blechová[1], Petr Kozlík[2] and Josef Cvačka[1,2*]

[1] Institute of Organic Chemistry and Biochemistry of the Czech Academy of Sciences, Prague, Czech Republic
[2] Department of Analytical Chemistry, Faculty of Science, Charles University, Prague, Czech Republic
* Correspondence: josef.cvacka@uochb.cas.cz; Tel.: (+420 220 183 303)

Figure S1. FTMS spectra of acetylated SLGE 2
Figure S2. MS^2 spectra of acetylated SLGE 3
Figure S3. MS^2 spectra of acetylated SLGE 4
Figure S4. FTMS spectra of acetylated SLGK 5
Figure S5. MS^2 spectra of acetylated SLGK. 6
Figure S6. FTMS spectra of acetylated VASLF 7
Figure S7. MS^2 spectra of acetylated VASLF 8
Figure S8. MS^2 spectra of acetylated VASLF 9
Figure S9. FTMS spectra of acetylated QTALVELLF 10
Figure S10. MS^2 spectra of acetylated QTALVELLF 11
Figure S11. FTMS spectra of acetylated AWSVAE 12
Figure S12. MS^2 spectra of acetylated AWSVAE 13
Figure S13. FTMS spectra of acetylated SLGF 14
Figure S14. MS^2 spectra of acetylated SLGF 15
Figure S15. FTMS spectra of acetylated VLASSAF 16
Figure S16. MS2 spectra of acetylated VLASSAF 17
Figure S17. FTMS spectra of acetylated QTALVELLE 18
Figure S18. MS2 spectra of acetylated QTALVELLE 19
Table S1. Peptides detected by APPI in BSA digested by chymotrypsin 20
Table S2. Peptides detected by APCI in BSA digested by chymotrypsin 21
Table S3. Peptides detected by ESI in BSA digested by chymotrypsin 22
Table S4. Peptides detected by APPI in BSA digested by Glu-C 23
Table S5. Peptides detected by APCI in BSA digested by Glu-C 24
Table S6. Peptides detected by ESI in BSA digested by Glu-C 25

A)

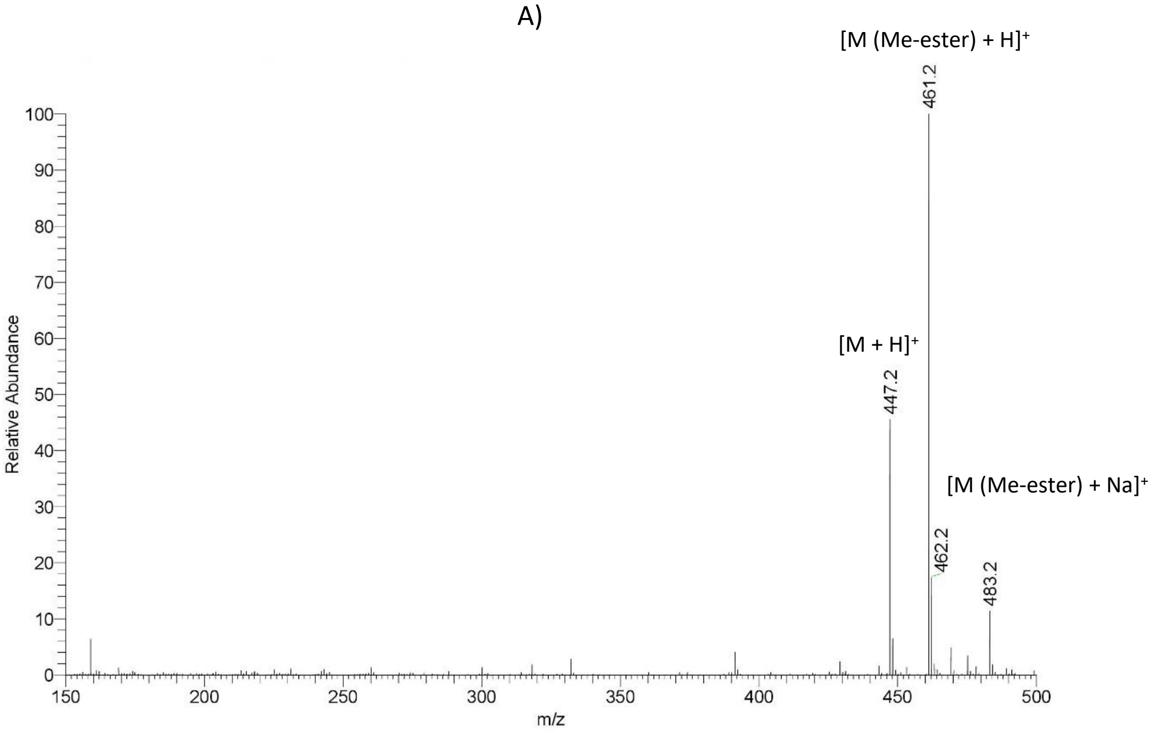

B)

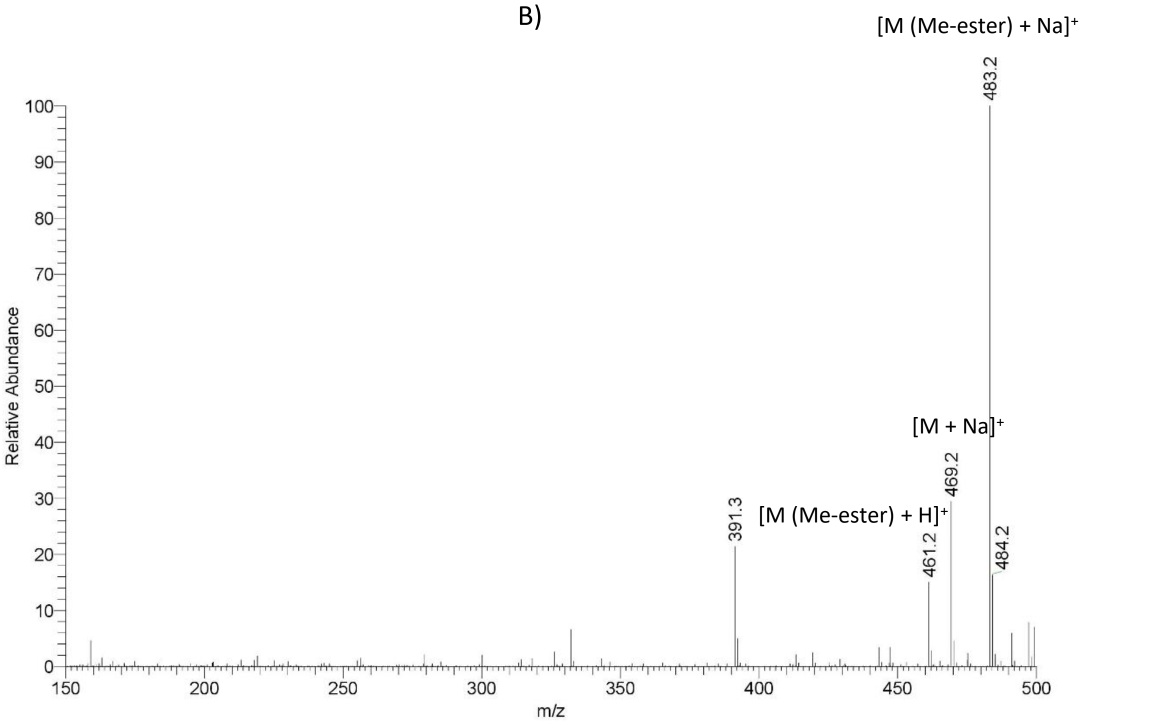

Figure S1. FTMS spectra of acetylated SLGE detected in A) APCI and B) APPI.

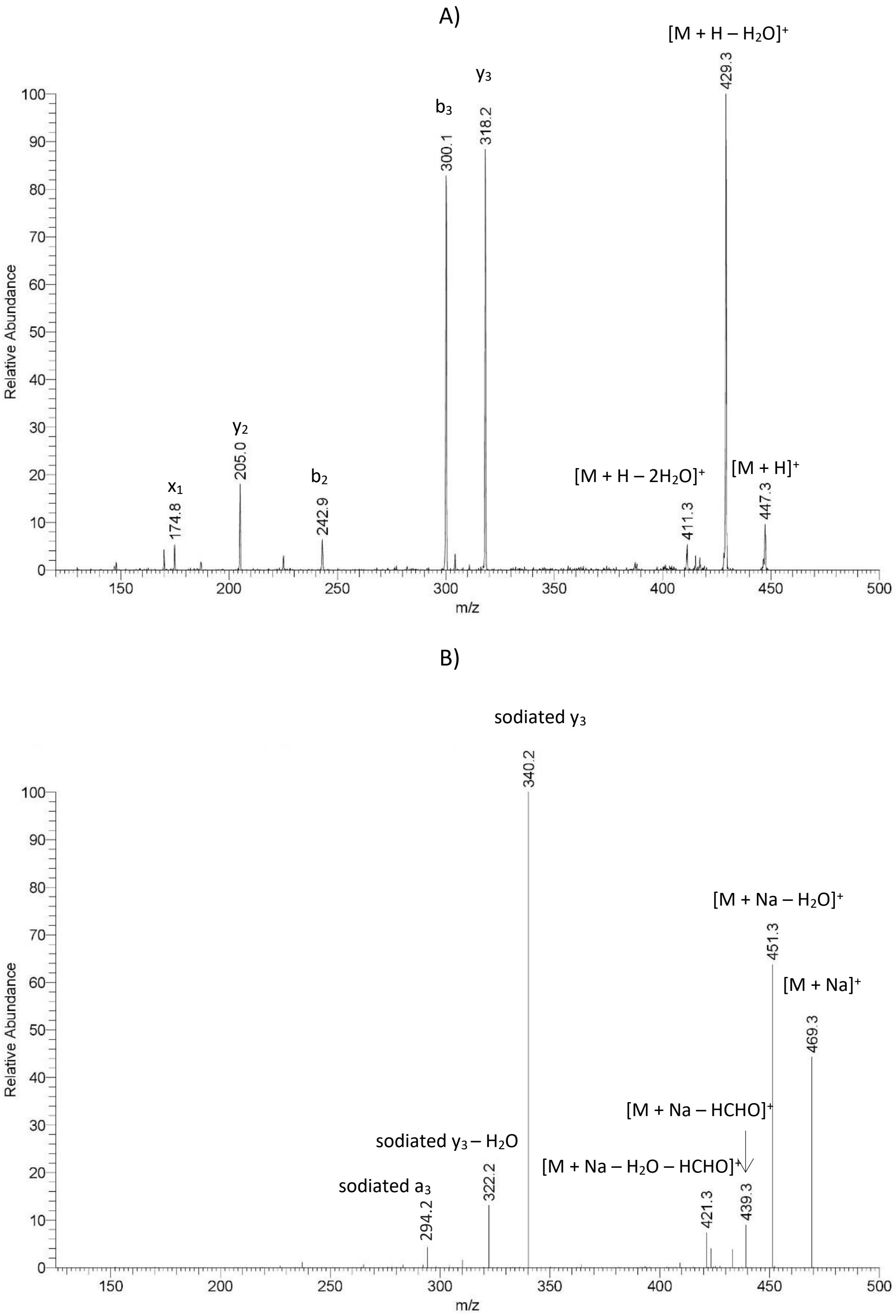

Figure S2. MS^2 spectra of acetylated SLGE A) precursor $[M + H]^+$ *m/z* 447.3, B) precursor $[M + Na]^+$ *m/z* 469.3.

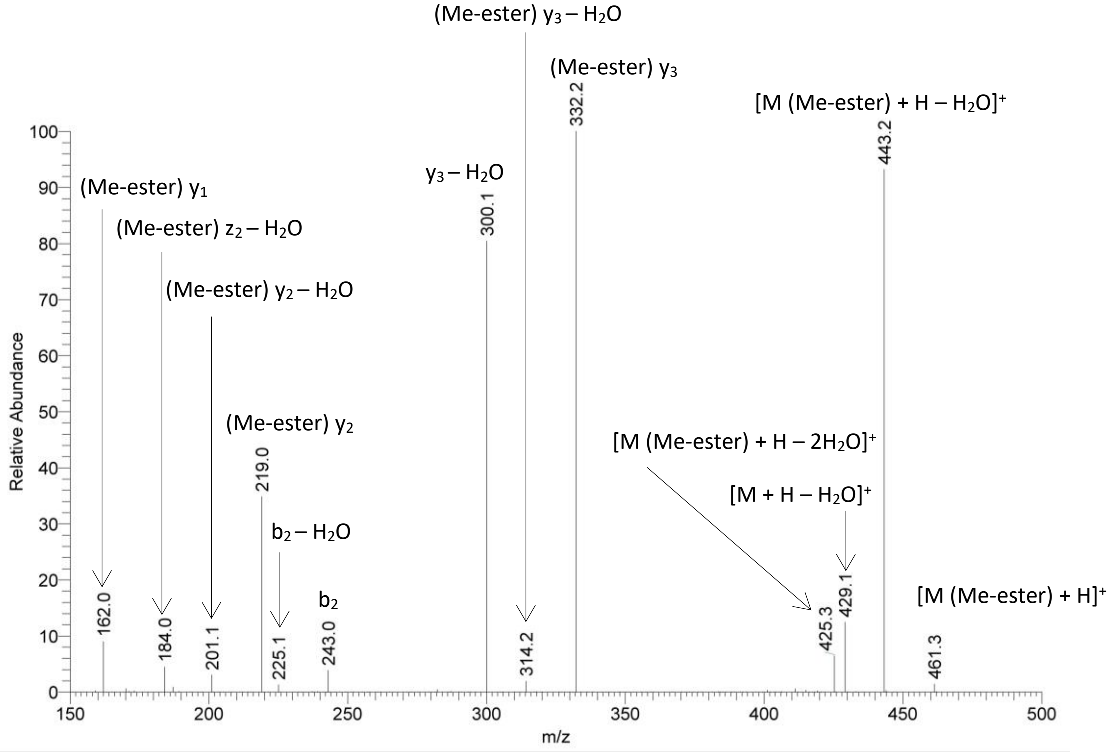

B)

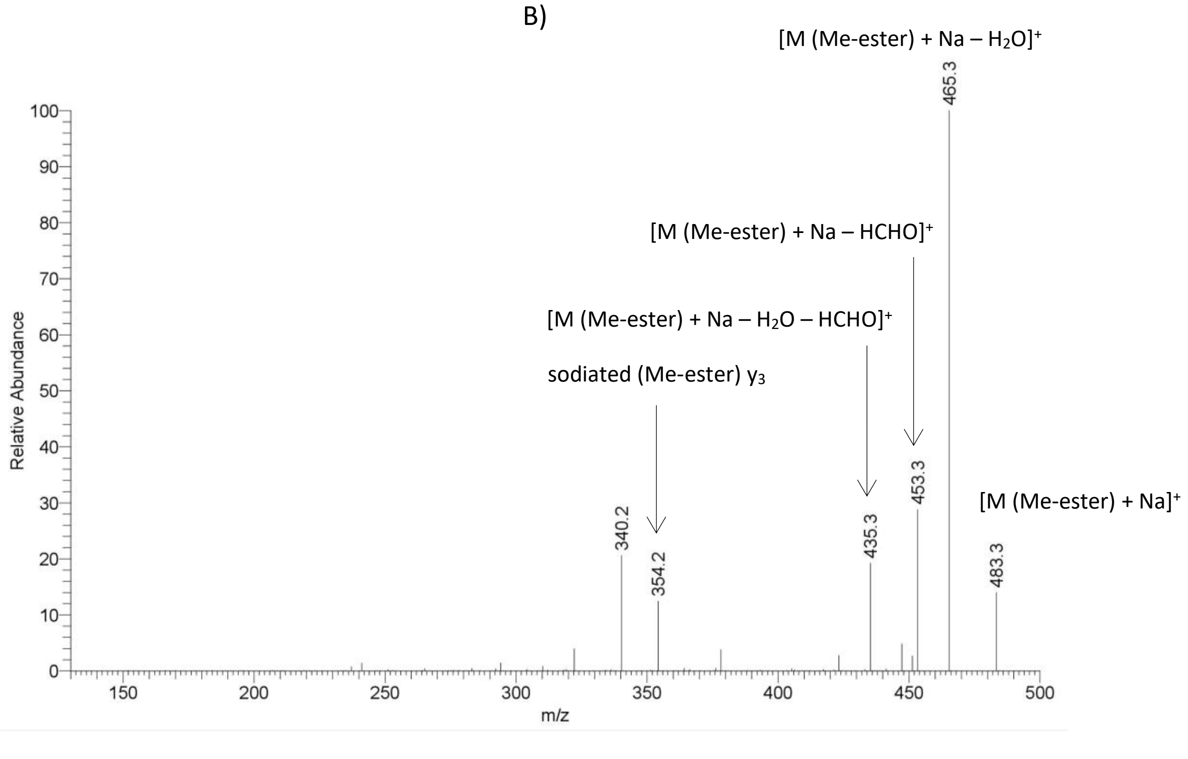

Figure S3. MS[2] spectra of acetylated SLGE A) precursor $[M$ (Me-ester) $+ H]^+$ *m/z* 461.3, B) $[M$ (Me-ester) $+ Na]^+$ *m/z* 483.3.

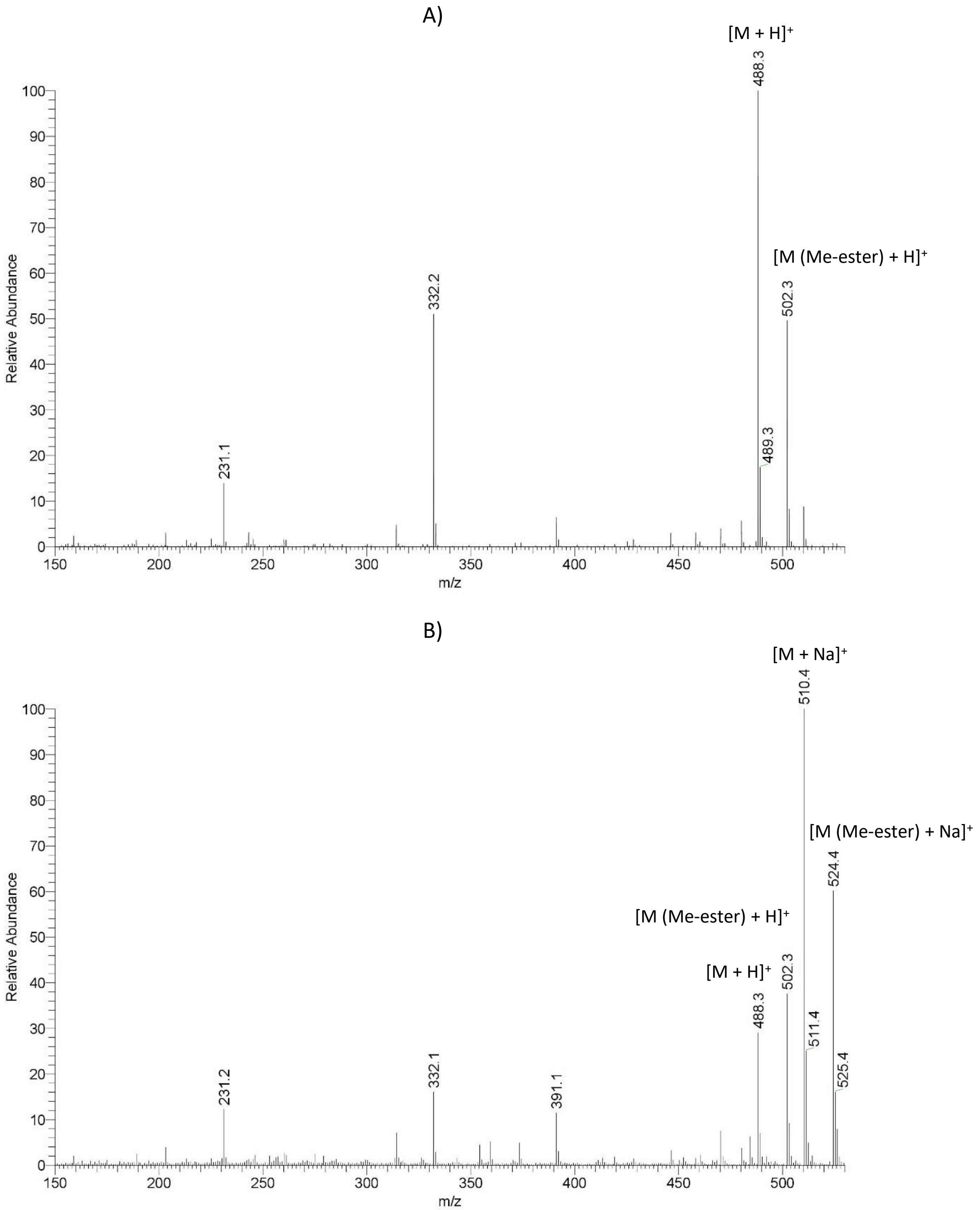

Figure S4. FTMS spectra of acetylated SLGK detected in A) APCI and B) APPI.

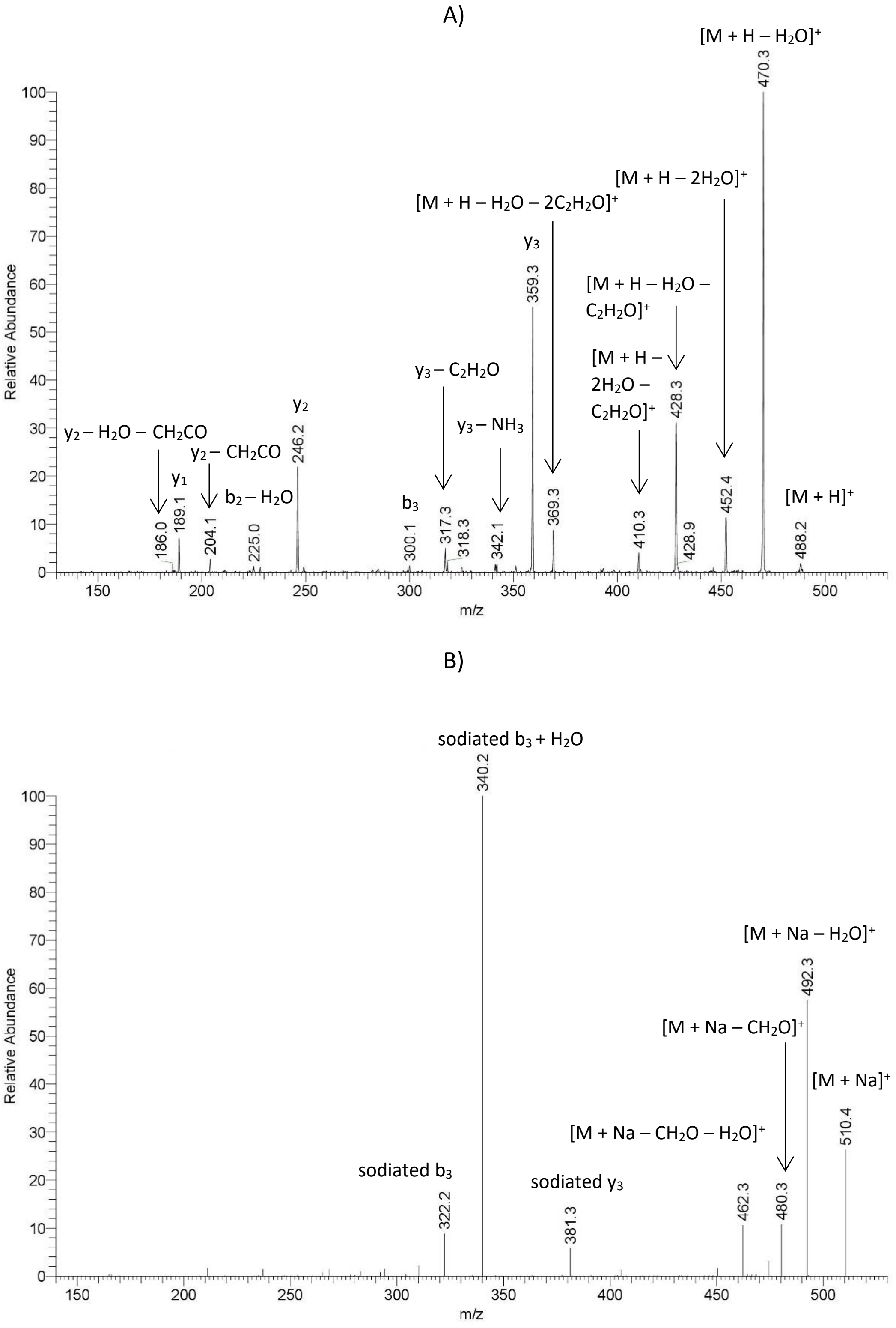

Figure S5. MS^2 spectra of acetylated SLGK A) precursor $[M + H]^+$ *m/z* 488.3, B) precursor $[M + Na]^+$ *m/z* 510.4.

A)

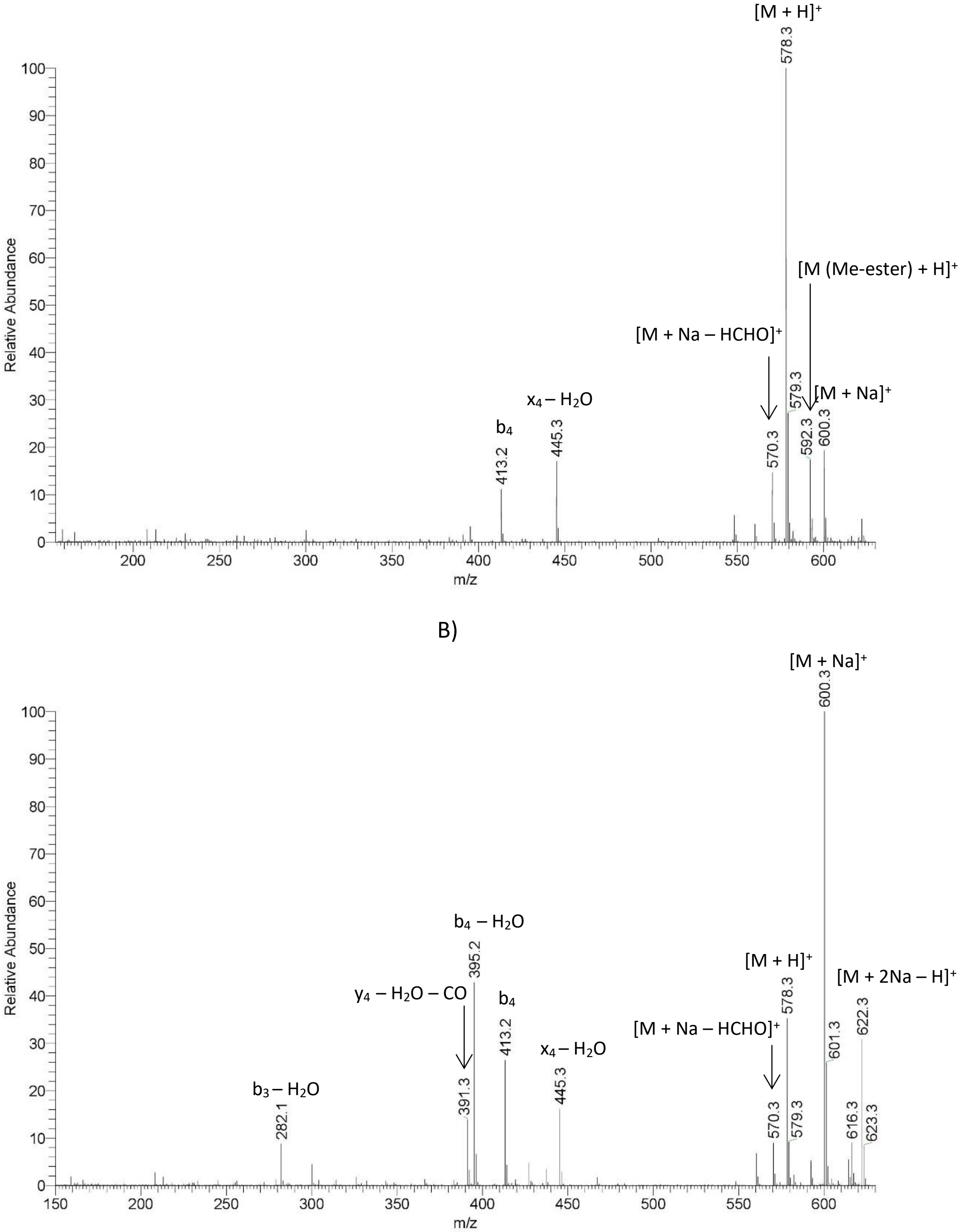

Figure S6. FTMS spectra of acetylated VASLF detected in A) APCI and B) APPI.

A)

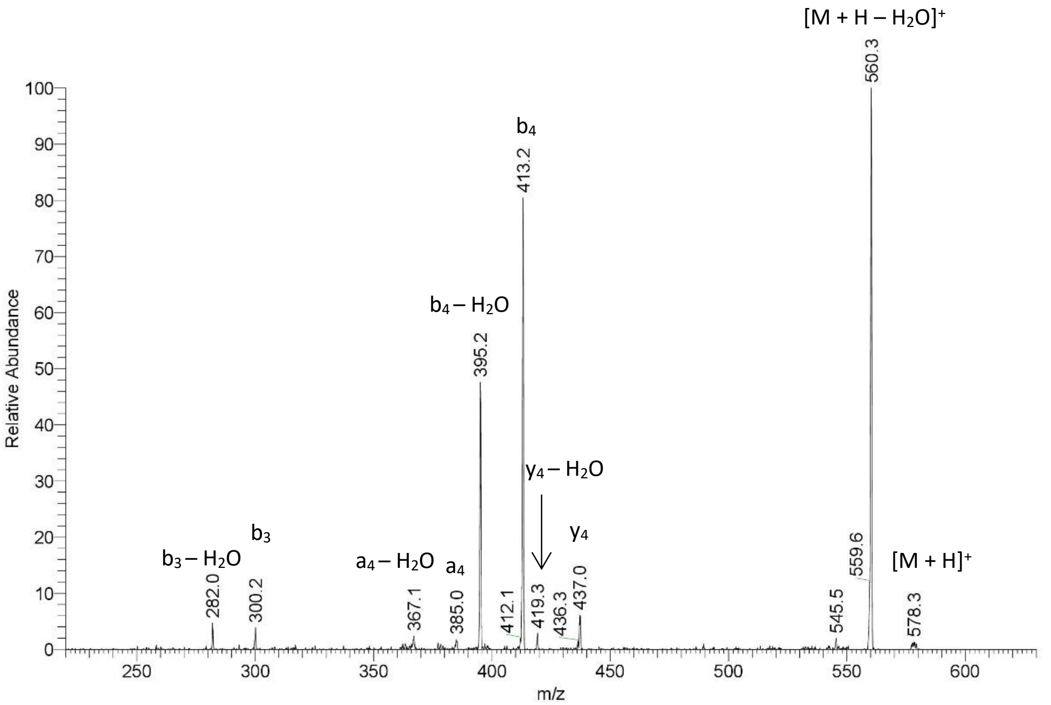

B)

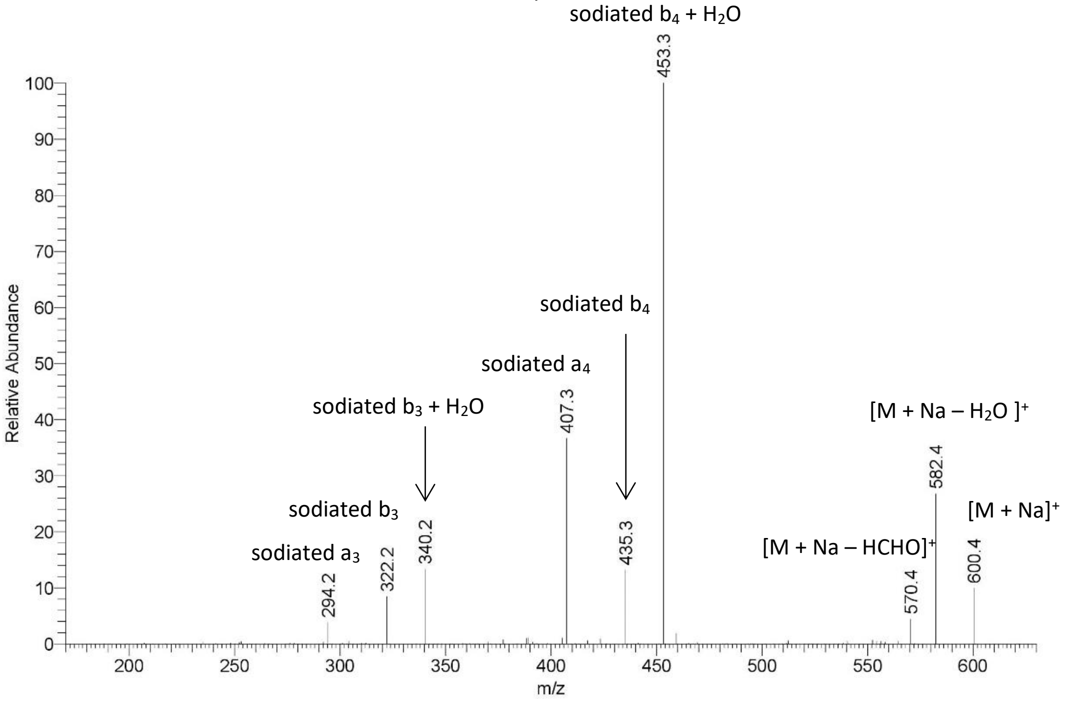

Figure S7. MS^2 spectra of acetylated VASLF A) precursor $[M + H]^+$ *m/z* 578.3, B) precursor $[M + Na]^+$ *m/z* 600.4.

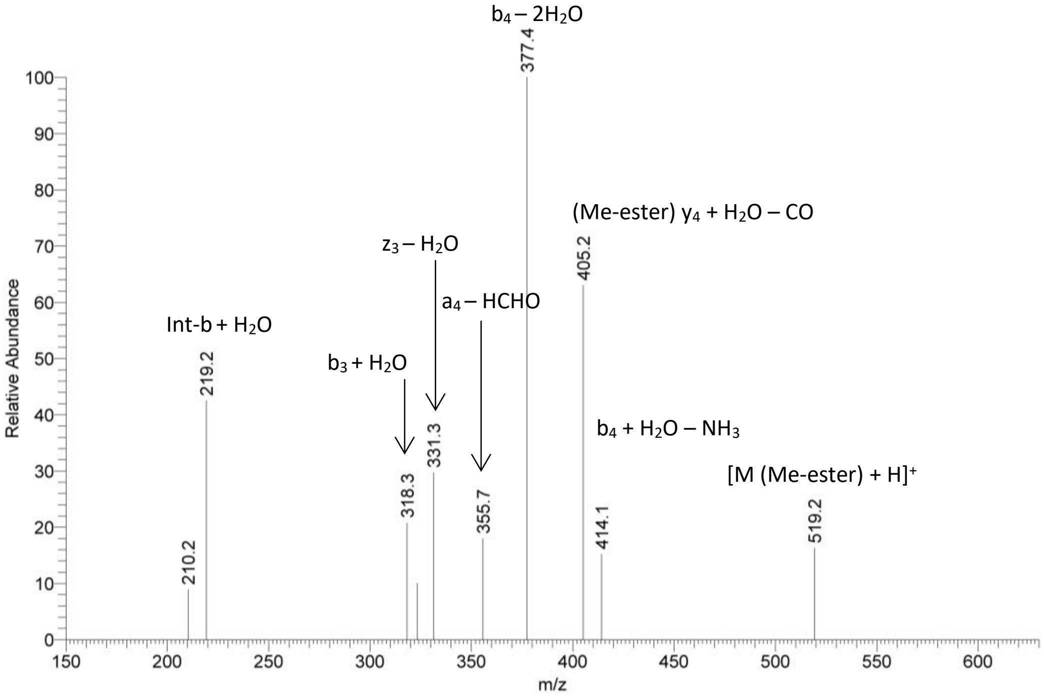

Figure S8. MS^2 spectra of acetylated VASLF precursor $[M\ (Me\text{-}ester) + H]^+$ *m/z* 519.2.

A)

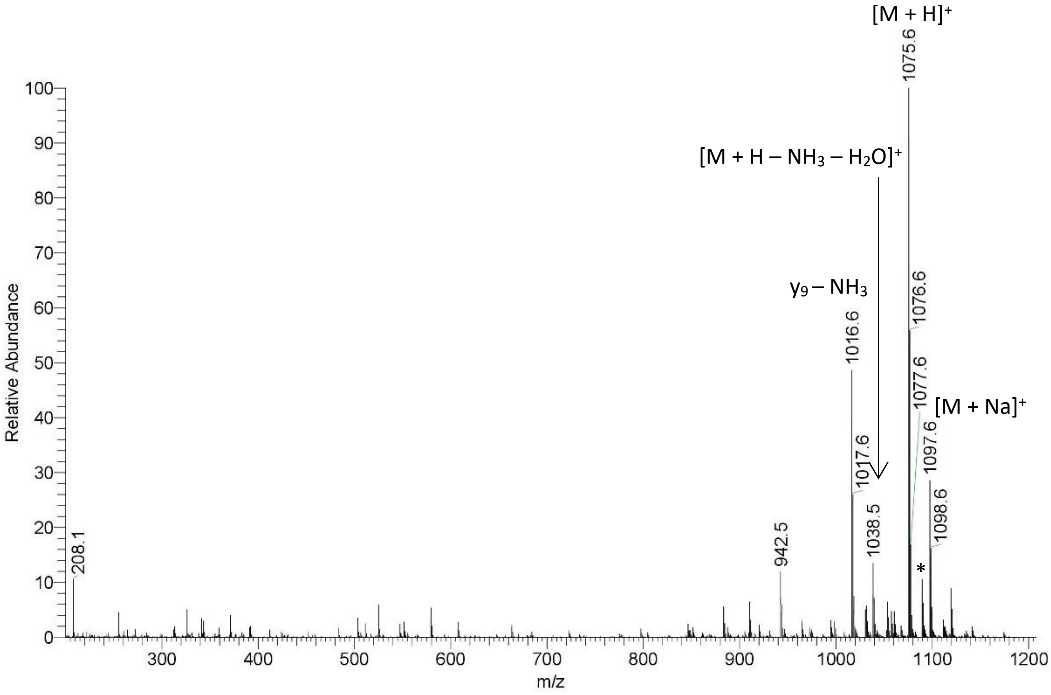

*m/z = 1089.6 [M (Me-ester) + H]+

B)

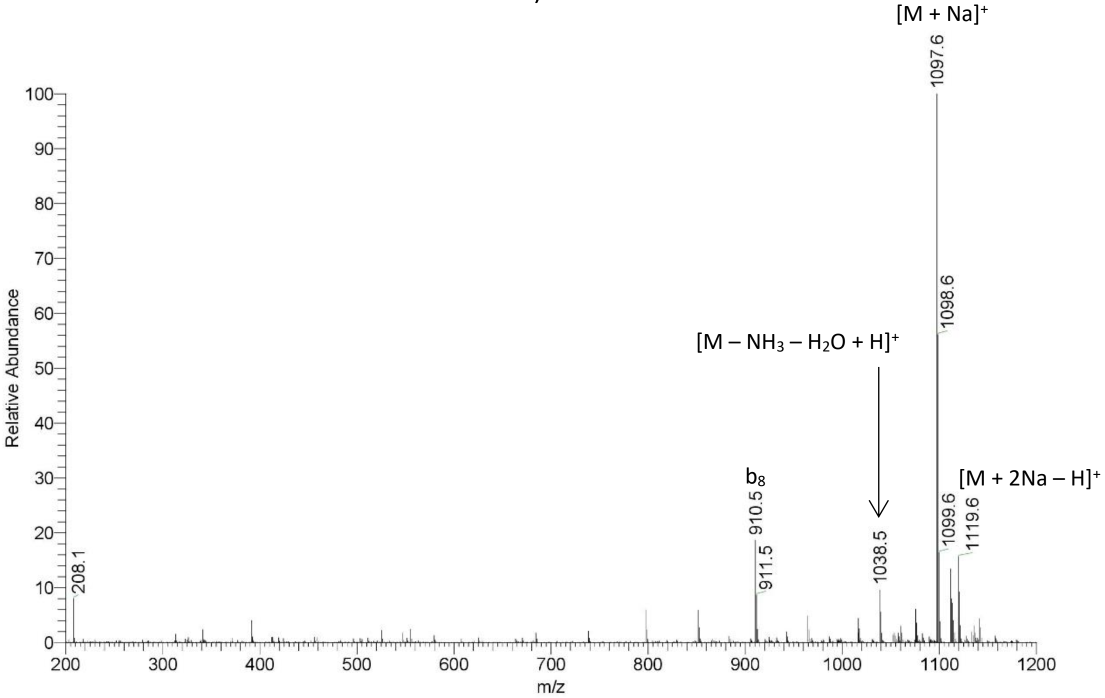

Figure S9. FTMS spectra of acetylated QTALVELLF detected in A) APCI and B) APPI.

A)

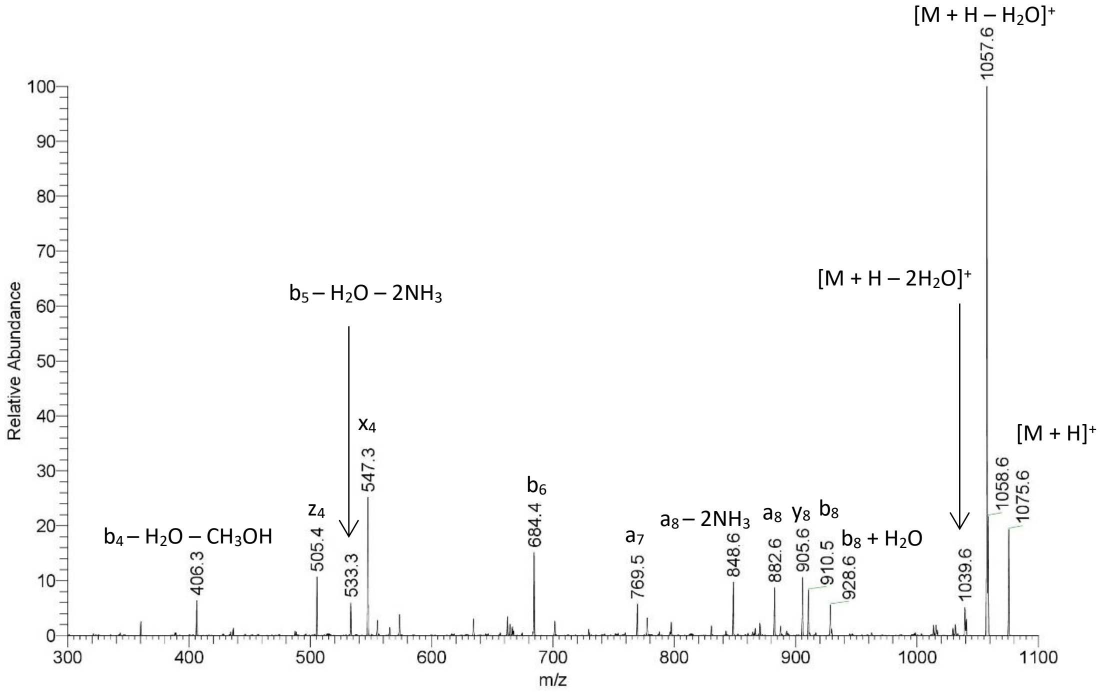

B)

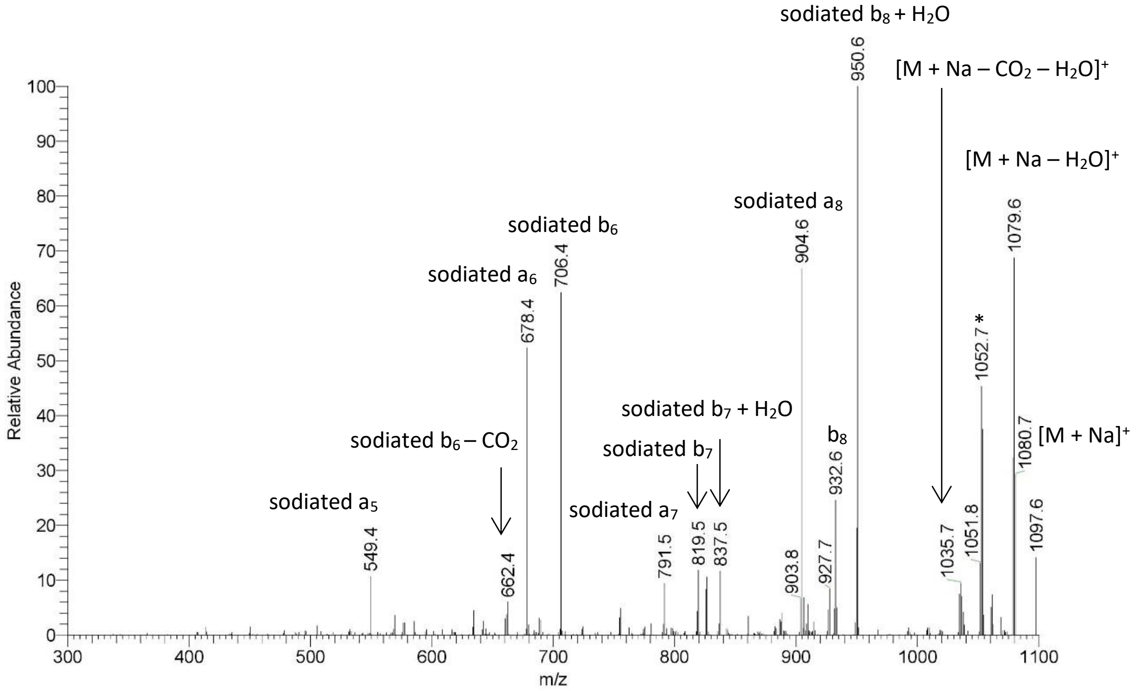

* m/z 1052.7 = [M + Na – CO – NH$_3$]$^+$

Figure S10. MS^2 spectra of acetylated QTALVELLF A) precursor [M + H]$^+$ *m/z* 1075.6, B) precursor [M + Na]$^+$ *m/z* 1097.6.

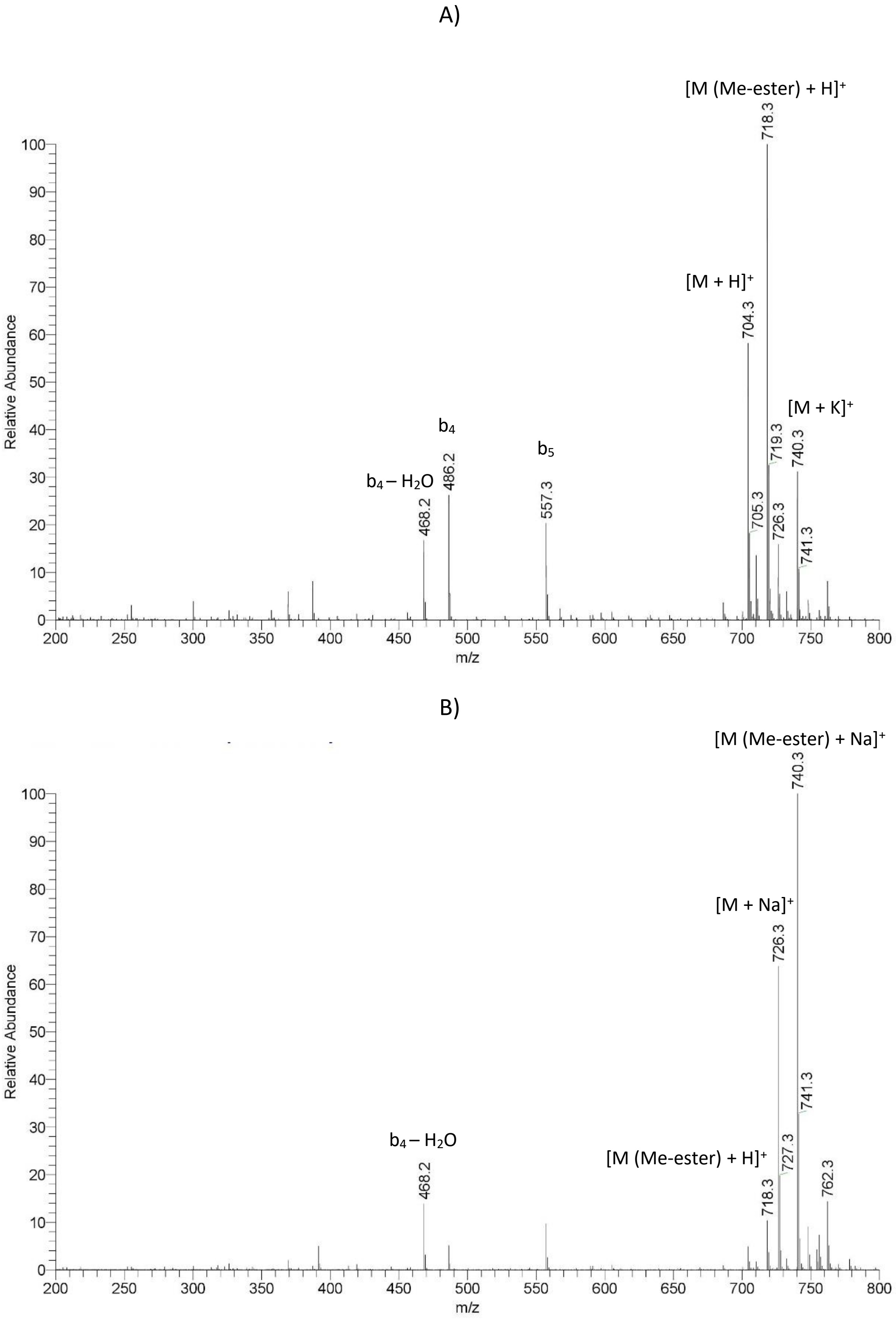

Figure S11. FTMS spectra of acetylated AWSVAE detected in A) APCI and B) APPI.

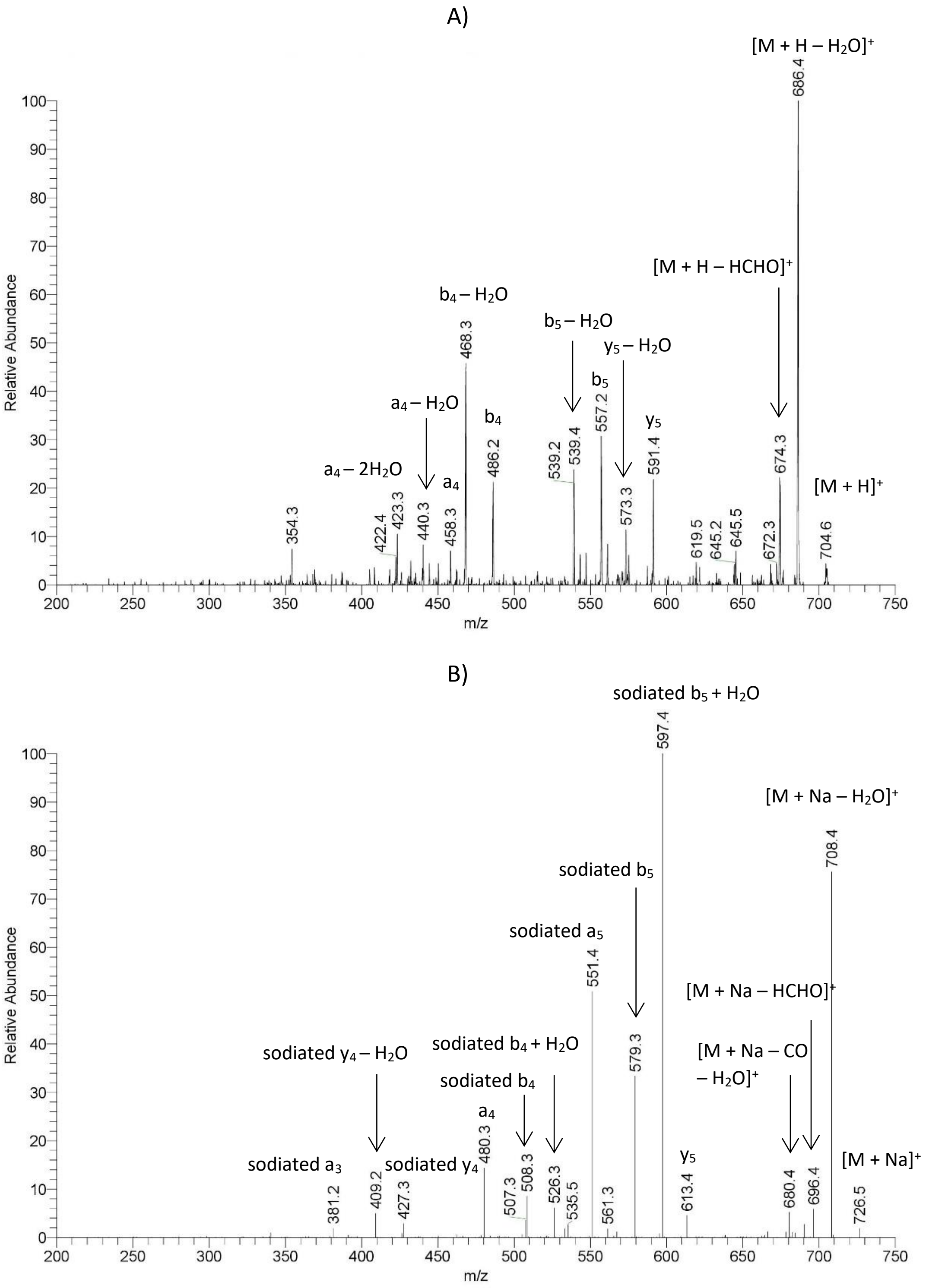

Figure S12. MS[2] spectra of acetylated AWSVAE A) precursor [M + H]+ *m/z* 704.6, B) precursor [M + Na]+ *m/z* 726.5.

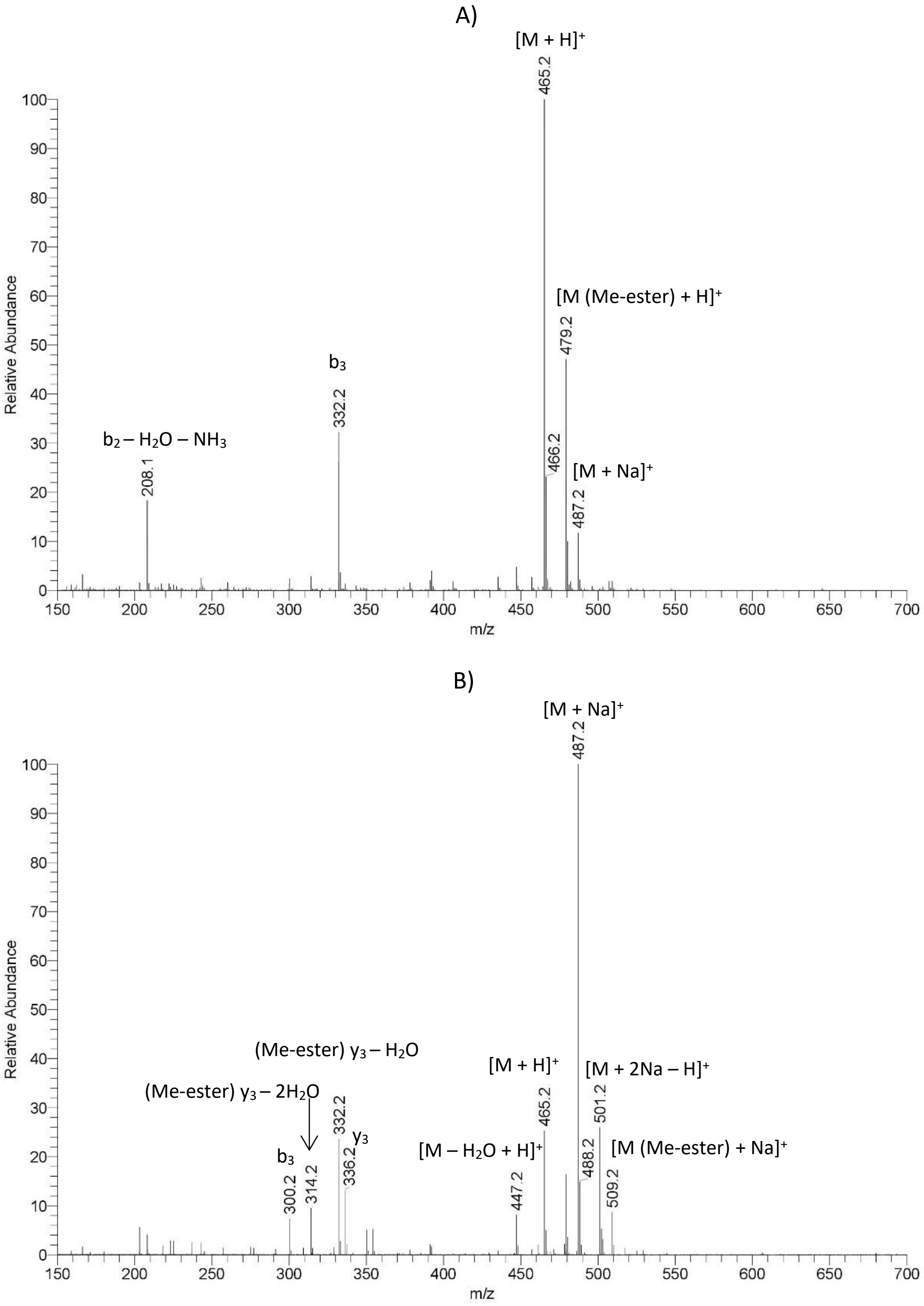

Figure S13. FTMS spectra of acetylated SLGF detected in A) APCI and B) APPI.

A)

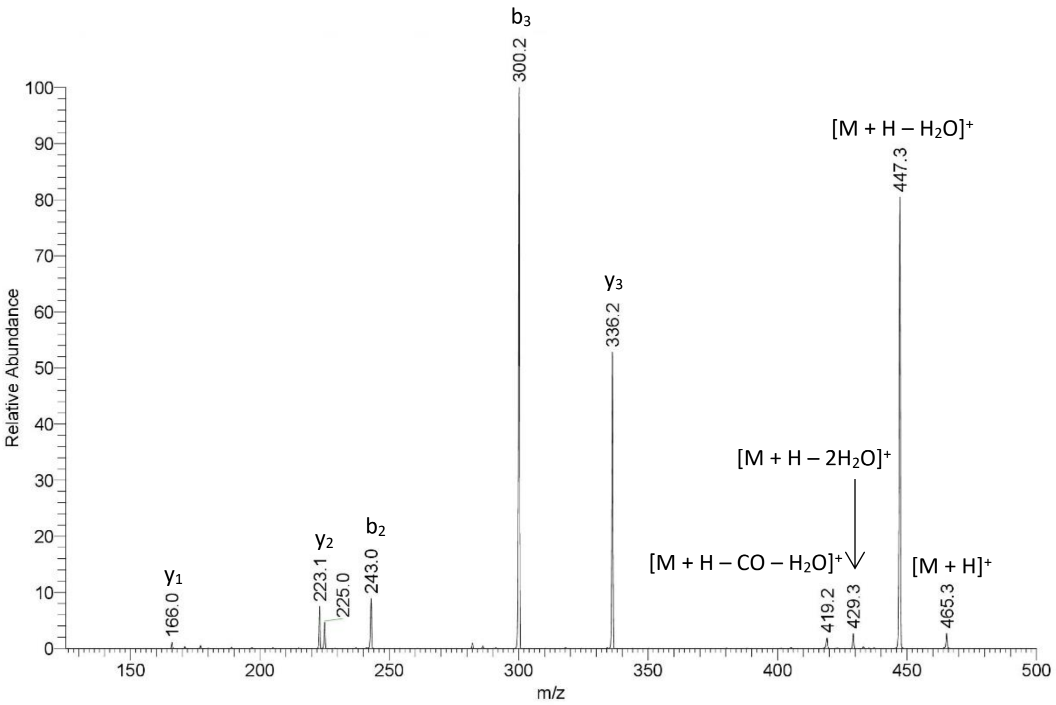

B)

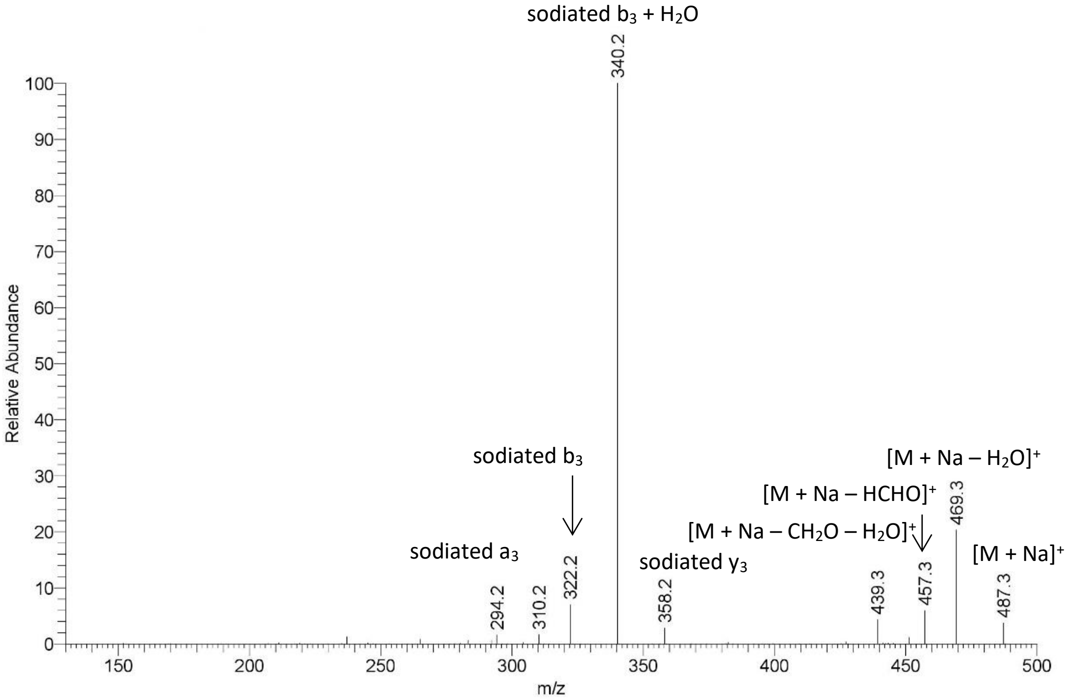

Figure S14. MS[2] spectra of acetylated SLGF A) precursor $[M + H]^+$ *m/z* 465.3, B) precursor $[M + Na]^+$ *m/z* 487.3.

A)

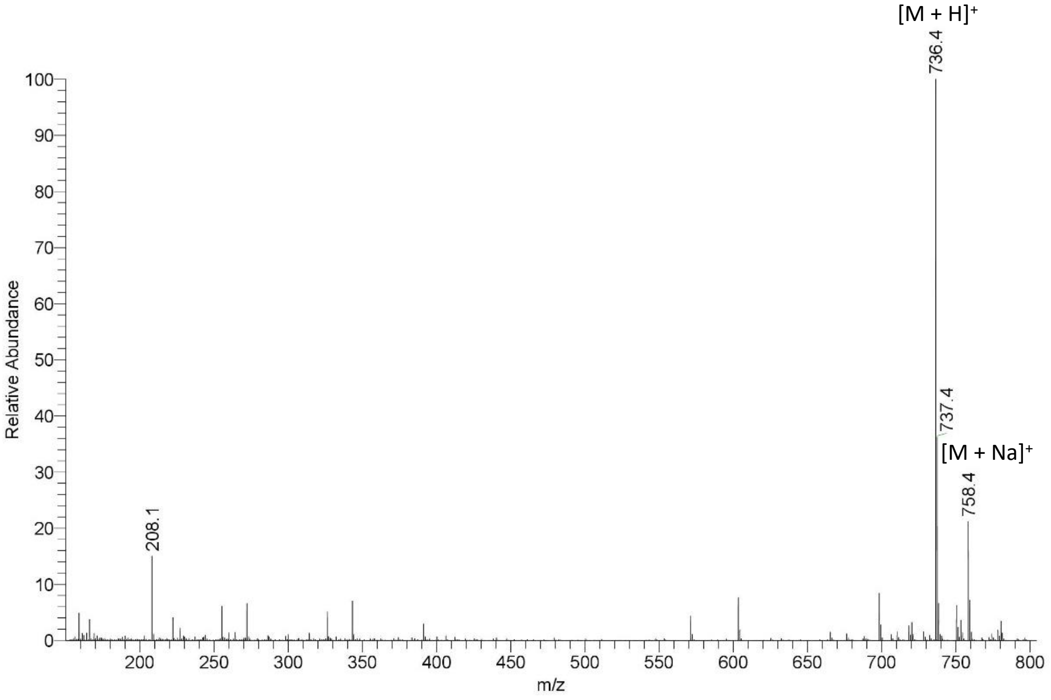

B)

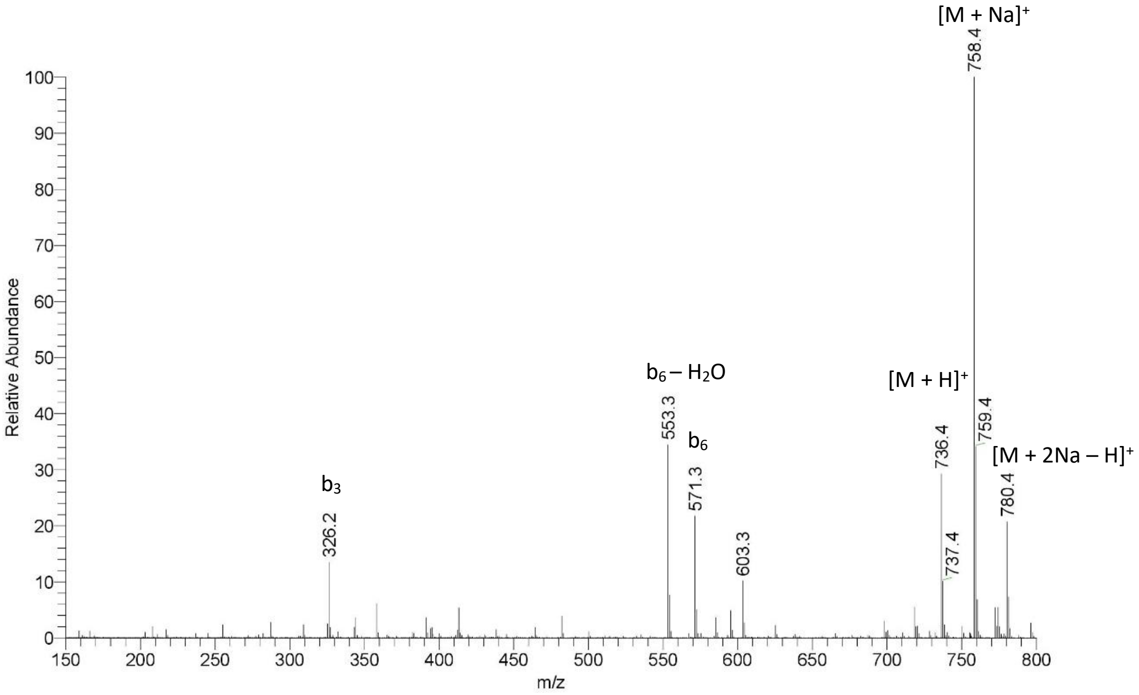

Figure S15. FTMS spectra of acetylated VLASSAF detected in A) APCI and B) APPI.

A)

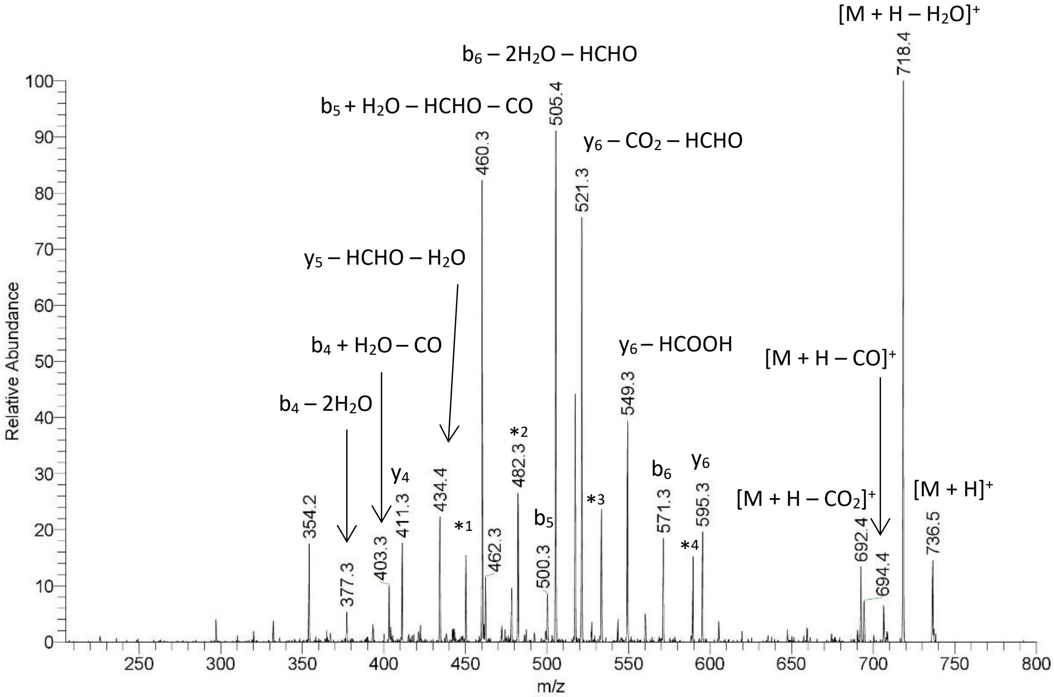

*1 m/z 450.3 = $y_5 – CH_3OH$

*2 m/z 482.3 = $y_5 – CO_2 – HCHO$

*3 m/z 533.3 = $b_6 + H_2O – C_4H_8$

*4 m/z 589.4 = $b_6 + H_2O$

B)

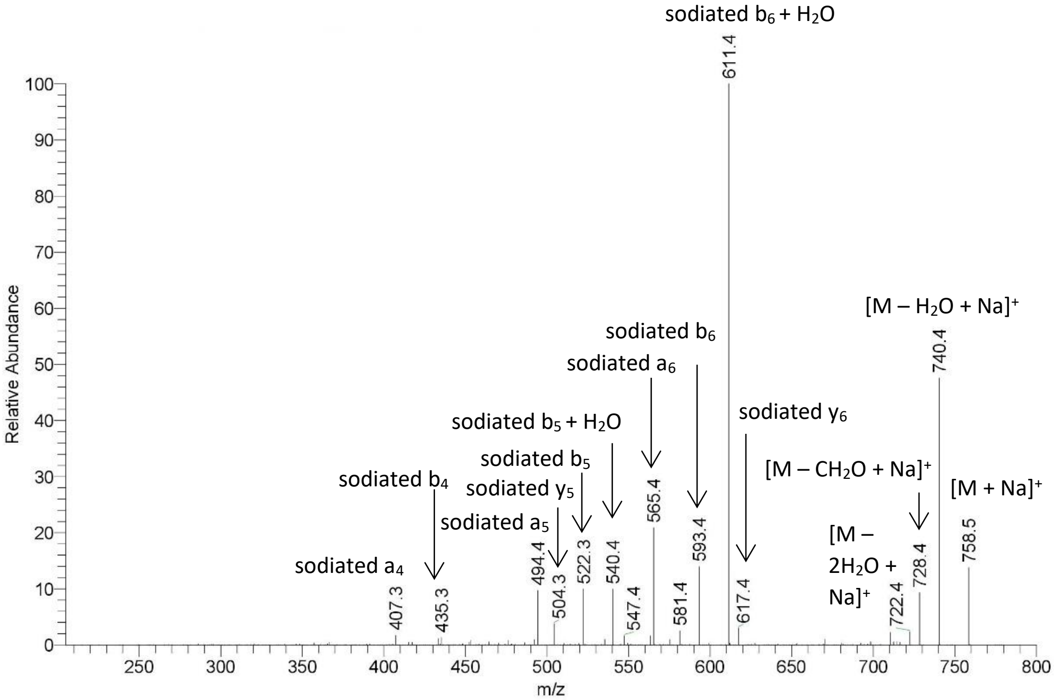

Figure S16. MS^2 spectra of acetylated VLASSAF A) precursor $[M + H]^+$ *m/z* 736.5, B) precursor $[M + Na]^+$ *m/z* 758.5.

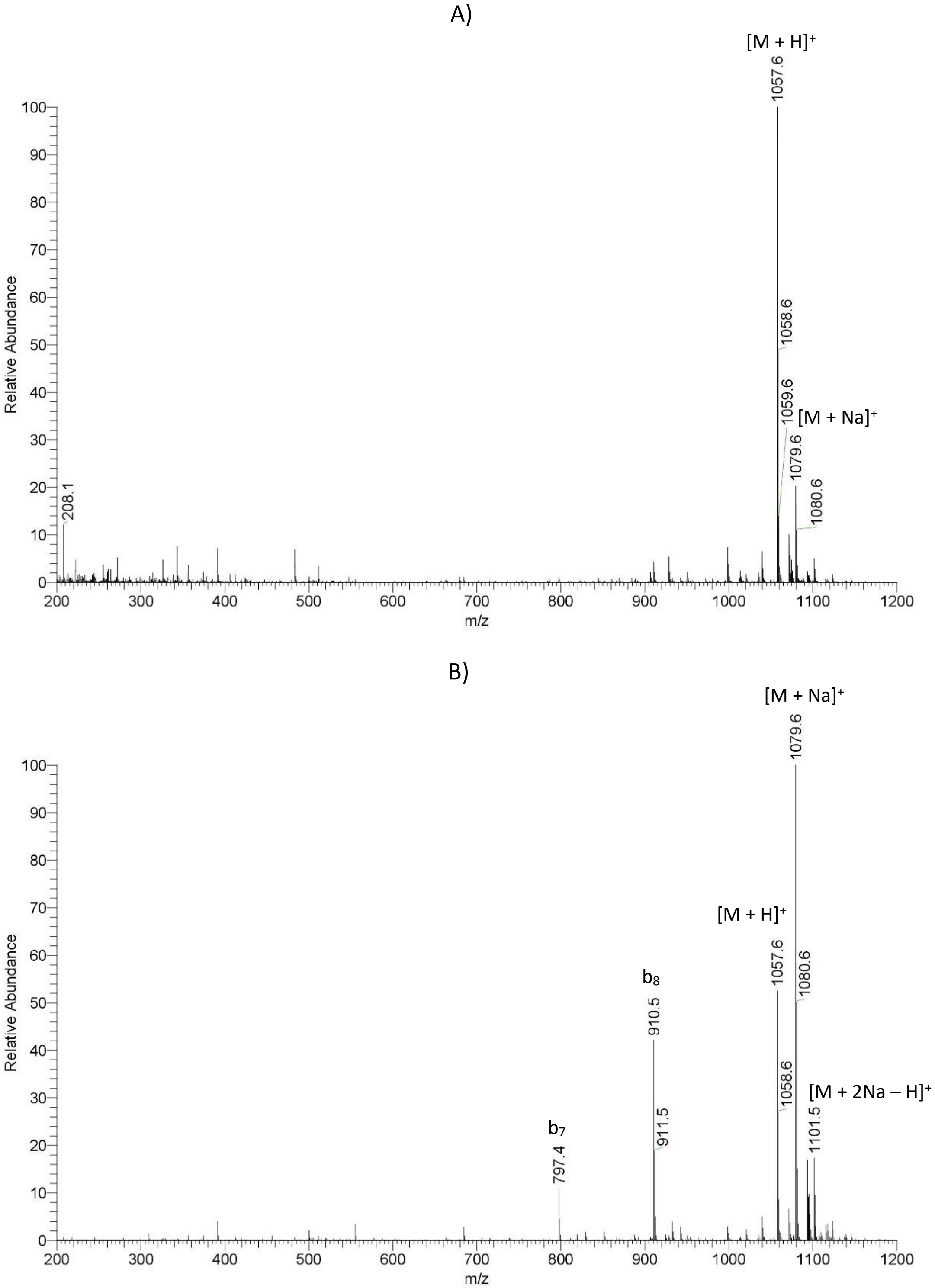

Figure S17. FTMS spectra of acetylated QTALVELLE detected in A) APCI and B) APPI.

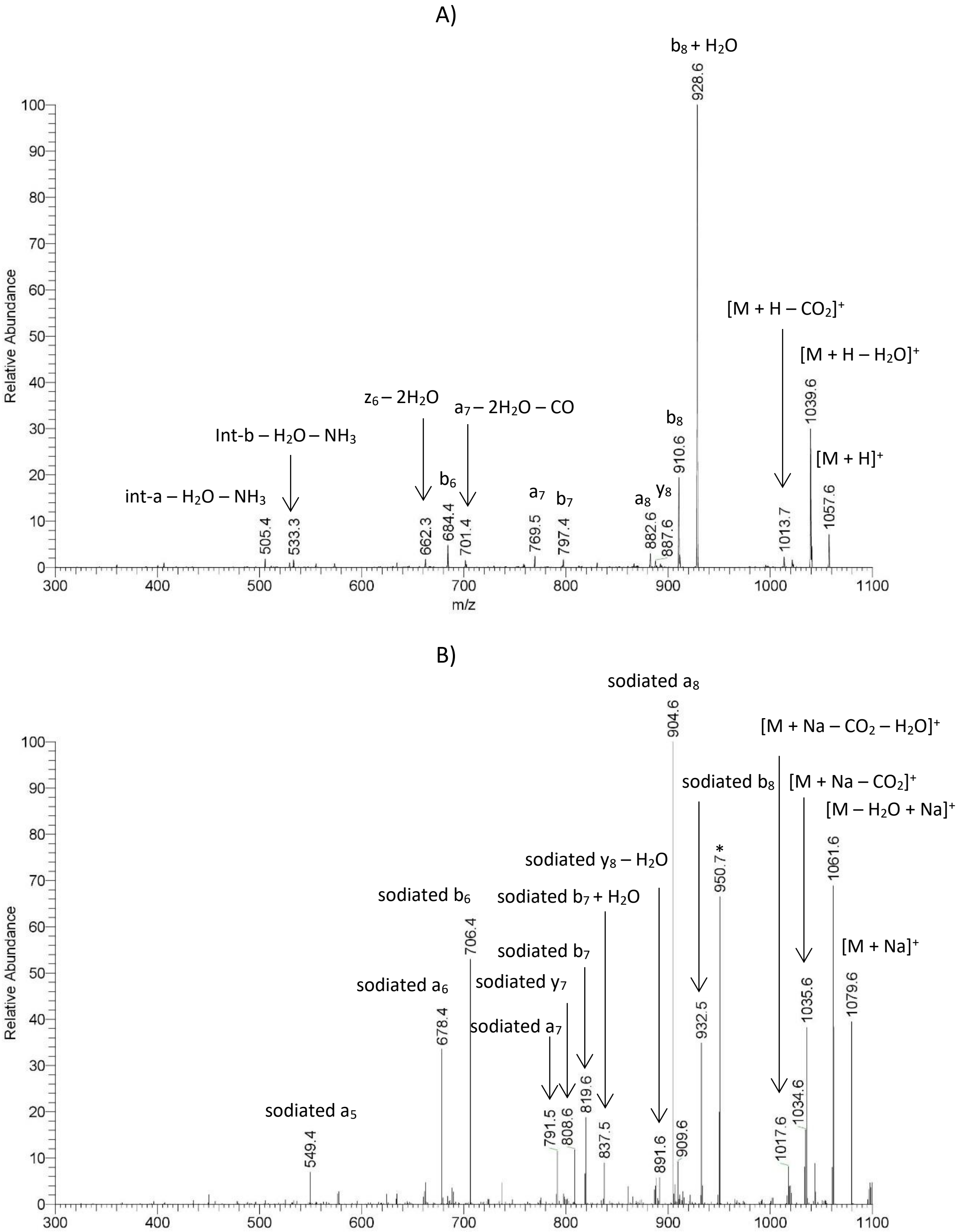

* m/z 950.7 = sodiated b_8

Figure S18. MS[2] spectra of acetylated QTALVELLE A) precursor $[M + H]^+$ *m/z* 1057.6, B) precursor $[M + Na]^+$ *m/z* 1079.6.

Table S1. Peptides detected by APPI in BSA digested by chymotrypsin. BSA was a) unmodified, or with b) acetylated ε amines of lysine side chain c) acetylated ε amines of lysine side chain together with peptides N-termini.

Peptides detected by APPI		
Non-acetylated	Acetylated ε amines of K	Acetylated K and N-termini
AKEY	DEHVKL	AKTCVADESHAGCEKSL
AKY	DKLKHLVDEPQNL	ANKYNGVF
AVSVL	EIARRHPY	AVEGPKL
DEHVKL	FLSHKDDSPDLPKL	AVEGPKLVVSTQTALA
DEKLF	GDMADCCEKQEPERNEC	DEHVKL
EKL	GERAL	DKL
GFQNAL	GFQNAL	DKLKHLVDEPQNL
IAFSQY	ILNRL	EIARRHPY
ILNRL	IVRY	GERAL
IVRY	KADEKKF	GFQNAL
KADEKKF	KADEKKFW	ILNRL
KAW	KDLGEEHF	IVRY
KGL	KECCDKPL	KADEKKF
KGLVL	LPKIETMREKVL	KAW
KTVMENF	LQQCPF	KDLGEEHF
KTVMENFVAF	LRLAKEY	KECCDKPL
LGSF	NRLCVL	KGLVL
LQQCPF	RCASIQKF	KHLVDEPQNL
QNAL	RLAKEY	LPKIETMREKVL
SQKFPKAEF	SQKFPKAEF	LQQCPF
STVF	SQKFPKAEFVEVTKL	LRLAKEY
SVARL	SVARL	RCASIQKF
VAF	TRKVPQVSTPTLVEVSRSL	RLAKEY
VELL	VEVTKL	SQKFPKAEF
VEVTKL	VNELTEF	SQKFPKAEFVEVTKL
VLIAF	VPKAFDEKLF	SVARL
VNELTEF	YEYSRRHPEY	TRKVPQVSTPTLVEVSRSL
VPKAF		VAF
WGKY		VELLKHKPKATE EQL
YANKY		VEVTKL
		VLIAF
		VNELTEF
		VPKAFDEKLF
		YANKY

Table S2. Peptides detected by APCI in BSA digested by chymotrypsin. BSA was a) unmodified, or with b) acetylated ε amines of lysine side chain c) acetylated ε amines of lysine side chain together with peptides N-termini.

Peptides detected by APCI		
Non-acetylated	Acetylated ε amines of K	Acetylated K and N-termini
AKEY	AKEY	AKEY
APELL	ANKY	ANKY
AVSVL	APELL	APELL
AVSVLL	APELLY	APELLY
DEHVKL	AVEGPKL	AVEGPKL
DEKLF	AVSVL	AVSVL
FYAPEL	AVSVLL	AVSVLL
GERAL	DEHVKL	DEKLF
GFQNAL	DEKLF	EKLGEY
IAFSQY	EKLGEY	GERAL
ILNRL	GERAL	GFQNAL
ISLL	GFQNAL	GSFL
IVRY	GSFL	IAFSQY
KADEKKFW	IAFSQY	ILNRL
KAW	ILNRL	IVRY
KGLVL	ISLLL	KADEKKF
KTVMENF	IVRY	KAW
KTVMENFVAF	KADEKKFW	KGLVL
LGSF	KAW	KPDPNTL
SQKFPKAEF	KGL	KTVMENF
STVF	KGLVL	LGSF
STVFDKL	KPDPNTL	LYEY
SVARL	KTVMENF	NGVF
VEVTKL	LGSF	SQKFPKAEF
VLIAF	LQQCPF	STVF
VNELTEF	LYEY	SVARL
VPKAF	MKWVTF	VELL
VPKAFDEKLF	NGVF	VEVTKL
WGKY	SQKFPKAEF	VLIAF
	STVF	VNELTEF
	STVFDKL	VPKAF
	SVARL	VPKAFDEKLF
	VELL	VTDL
	VEVTKL	WGKY
	VNELTEF	YANKY
	VPKAF	YAPELL
	VPKAFDEKLF	
	VTDL	
	WGKY	
	YANKY	
	YAPELL	

Table S3. Peptides detected by ESI in BSA digested by chymotrypsin. BSA was a) unmodified, or with b) acetylated ε amines of lysine side chain c) acetylated ε amines of lysine side chain together with peptides N-termini.

Peptides detected by ESI		
Non-acetylated	Acetylated ε amines of K	Acetylated K and N-termini
AEDKDVCKNY	AEDKDVCKNY	ANKY
AKEY	CKVASL	AVEGPKLVVSTQTALA
AVEGPKLVVSTQTALA	DKLKHLVDEPQNL	AVSVL
DEHVKL	EATLEECCAKDDPHACY	AVSVLL
DEHVKLVNELTEF	GFQNAL	DEHVKL
DEKLF	ICDNQDTISSKL	DEKLF
DKLKHLVDEPQNL	IKQNCDQFEKLGEY	DKLKHLVDEPQNL
GERAL	KADEK KFW	EATLEECCAKDDPHACY
GFQNAL	KDLGEEHF	GERAL
GFQNALIVRY	KECCDKPL	GFQNAL
ICDNQDTISSKL	KGLVL	IAFSQY
IKQNCDQF	KHKPKATEEQL	IKQNCDQFEKLGEY
IKQNCDQFEKLGEY	KHKPKATEEQLKTVMENF	KADEKKF
ILNRL	KTVMENF	KDLGEEHF
IVRY	LPKIETMREKVL	KGLVL
KDLGEEHF	LQQCPF	KHKPKATEEQL
KDLGEEHFKGL	LRLAKEY	KHKPKATEEQLKTVMENF
KHKPKATEEQL	NGVFQECCQAEDKGACL	KHKPKATEEQLKTVMENFVAF
KTVMENFVAF	QEAKDAF	KTVMENFVAF
LGSF	RCASIQKF	LGSF
LQQCPFDEHVKL	SALTPDETYVPKAF	LKHKPKATEEQLKTVMENF
NGVFQECCQAEDKGACL	SQKFPKAEF	LQQCPF
QEAKDAF	VDKCCAADDKEACF	LQQCPFDEHVKL
QEAKDAFLGSF	VEVTKL	LRLAKEY
QECCQAEDKGACL	VLIAF	QEAKDAF
RLAKEY	VNELTEF	QECCQAEDKGACL
SALTPDETYVPKAF	VPKAFDEKLF	RCASIQKF
SQKFPKAEF		RLAKEY
TPDETY		SQKFPKAEF
VDKCCAADDKEACF		SQKFPKAEFVEVTKL
VEVTKLVTDL		STVF
VLIAF		SVARL
VNELTEF		TRKVPQVSTPTLVEVSRSL
VPKAF		VEVTKL
VPKAFDEKLF		VEVTKLVTDL
		VLIAF
		VNELTEF
		VPKAF
		VPKAFDEKLF
		WGKY

Table S4. Peptides detected by APPI in BSA digested by Glu-C. BSA was a) unmodified, or with b) acetylated ε amines of lysine side chain c) acetylated ε amines of lysine side chain together with peptides N-termini.

Peptides detected by APPI		
Non-acetylated	Acetylated ε amines of K	Acetylated K and N-termini
ACFAVE	ACFAVE	ACFAVE
AFLGSFLYE	AFLGSFLYE	AFLGSFLYE
AKDAFLGSFLYE	CADDRAD	AKDAFLGSFLYE
CCDKPLLE	CCAKD D	CCDKPLLE
DKGACLLPKIE	CCDKPLLE	CCQAE
FAKTCVADE	DKE	CCHGDLLE
FKADE	DKGACLLPKIE	DKEACFAVE
GPKLVVSTQTALA	DRAD	DKGACLLPKIE
HVKLVNE	DYLSLILNRLCVLHE	DYLSLILNRLCVLHE
IAHRFKDLGEE	EQLKTVME	EQLKTVME
IARRHPYFYAPE	ETYVPKAFD	ETYVPKAFD
ICTLPDTE	FAKTCVADE	FAKTCVADE
KDAIPE	FVE	FKADE
KGACLLPKIE	GPKLVVSTQTALA	GPKLVVSTQTALA
KKFWGKYLYE	HVKLVNE	HVKLVNE
KLFTFHAD	IAHRFKD	IAHRFKD
KLGE	IAHRFKDLGEE	IAHRFKDLGEE
KPLLE	IARRHPYFYAPE	IARRHPYFYAPE
KQEPE	ICTLPDTE	ICTLPDTE
KQEPERNE	KDAIPE	KDAIPE
KQIKKQTALVE	KGACLLPKIE	KKFWGKYLYE
KSHCIAE	KLFTFHAD	KLFTFHAD
KSLHTLFGDE	KLGE	KQEPE
KTPVSE	KPLLE	KQEPERNE
KVTKCCTE	KSHCIAE	KSHCIAE
LCKVASLRE	KSLHTLFGDE	KSLHTLFGDE
LLKHKPKATE	KTPVSE	KTPVSE
LLKHKPKATEE	LCKVASLRE	LCKVASLRE
LTKVHKE	LLKHKPKATE	LLKHKPKATE
NLPPLTADFAE	LLKHKPKATEE	LLKHKPKATEE
QLKTVME	LTKVHKE	LLYYANKYNGVFQE
SHAGCE	NFVAFVD	LTKVHKE
TYVPKAFDE	NLPPLTADFAE	NLPPLTADFAE
VEKDAIPE	QFEKLGEYGFQNALIVRYTRKVPQVSTPTLVE	NQDTISSKLKE
VTKLVTD	RALKAWSVARLSQKFPKAE	QLKTVME
VTKLVTDLTKVHKE	RMPCTE	RALKAWSVARLSQKFPKAE
	RNE	RMPCTE
	VEKDAIPE	SERMPCTE
	VSRSLGKVGTRCCTKPESE	SHAGCE
	VTKLVTD	SLVNRRPCFSALTPDE
	YAVSVLLRLAKE	TEKQIKKQTALVE
	YGFQNALIVRYTRKVPQVSTPTLVE	TYVPKAFDE
	YLSLILNRLCVLHEKTPVSE	VEKDAIPE
	YSRRHPE	VTKLVTD
		YAVSVLLRLAKE
		YEATLE

		YGFQNALIVRYTRKVPQVSTPTLVE

Table S5. Peptides detected by APCI in BSA digested by Glu-C. BSA was a) unmodified, or with b) acetylated ε amines of lysine side chain c) acetylated ε amines of lysine side chain together with peptides N-termini.

Peptides detected by APCI		
Non-acetylated	Acetylated ε amines of K	Acetylated K and N-termini
ACFAVE	ACFAVE	ACFAVE
AFLGSFLYE	AFLGSFLYE	AFLGSFLYE
AKDAFLGSFLYE	ATLE	ATLE
ATLE	FKADE	DKEACFAVE
CCQAE	FVE	EHVKLVNE
DKGACLLPKIE	GPKLVVSTQTALA	EQLKTVME
FKADE	HVKLVNE	FKADE
GPKLVVSTQTALA	ICTLPDTE	GPKLVVSTQTALA
KGACLLPKIE	KDAIPE	HVKLVNE
KKFWGKYLYE	KGACLLPKIE	ICTLPDTE
KLGE	KLGE	KDAIPE
KQEPE	KPLLE	KLFTFHAD
KQIKKQTALVE	KQEPE	KLGE
KTPVSE	KSHCIAE	KSHCIAE
LLKHKPKATE	KTPVSE	KSLHTLFGDE
LLKHKPKATEE	LAKYICD	KTPVSE
LTKVHKE	LGEE	LCKVASLRE
QLKTVME	LLKHKPKATE	LTKVHKE
TISSKLKE	LTKVHKE	NLPPLTADFAE
TYVPKAFDE	NLPPLTADFAE	QLKTVME
VTKLVTD	VTKLVTD	SLVNRRPCFSALTPDE
	YEATLE	VTKLVTD
		YAVSVLLRLAKE

Table S6. Peptides detected by ESI in BSA digested by Glu-C. BSA was a) unmodified, or with b) acetylated ε amines of lysine side chain c) acetylated ε amines of lysine side chain together with peptides N-termini.

Peptides detected by ESI		
Non-acetylated	Acetylated ε amines of K	Acetylated K and N-termini
ACFAVEGPKLVVSTQTALA	ACFAVE	ACFAVE
AKDAFLGSFLYE	AFLGSFLYE	AKDAFLGSFLYE
ATLEE	CCDKPLLE	ATLEE
CCAKDDPHACYSTVFD	DKGACLLPKIE	CCDKPLLE
CCQAE	FAKTCVADE	CCQAE
CCHGDLLE	HVKLVNE	CCHGDLLE
DKDVCKNYQE	IARRHPYFYAPE	DKGACLLPKIE
EQLKTVME	ICTLPDTE	DYLSLILNRLCVLHE
ETYVPKAFDE	KLFTFHAD	FAKTCVADE
FAKTCVADE	KLGE	FKADE
FAKTCVADESHAGCE	KQIKKQTALVE	GPKLVVSTQTALA
FKADE	KSHCIAE	HVKLVNE
HVKLVNE	KTPVSE	IAHRFKD
KDAIPE	KVTKCCTE	IAHRFKDLGEE
KLGE	LCKVASLRE	IARRHPYFYAPE
KQEPE	LLKHKPKATE	KDAIPE
KQEPERNE	LLKHKPKATEE	KKFWGKYLYE
KSHCIAE	NLPPLTADFAE	KLFTFHAD
KVTKCCTE	QLKTVME	KLGE
LCKVASLRE	RALKAWSVARLSQKFPKAE	KQEPE
LLKHKPKATE	RMPCTE	KQIKKQTALVE
LLYYANKYNGVFQE	SLVNRRPCFSALTPDE	KSHCIAE
LTKVHKE	TYVPKAFDE	KTPVSE
QLKTVME	VSRSLGKVGTRCCTKPE	KVTKCCTE
RMPCTE	VTKLVTD	LCKVASLRE
SERMPCTE		LLKHKPKATE
SHAGCE		LLKHKPKATEE
TYGDMADCCE		LLYYANKYNGVFQE
VEKDAIPE		QLKTVME
YAVSVLLRLAKEYE		RMPCTE
		TYVPKAFDE
		VEKDAIPE
		VTKLVTD
		YAVSVLLRLAKE
